# *Spinacia oleracea* extract attenuates disease progression and sub-chondral bone changes in monosodium iodoacetate-induced osteoarthritis in rats

**DOI:** 10.1186/s12906-018-2117-9

**Published:** 2018-02-20

**Authors:** Dharmendra Choudhary, Priyanka Kothari, Ashish Kumar Tripathi, Sonu Singh, Sulekha Adhikary, Naseer Ahmad, Sudhir Kumar, Kapil Dev, Vijay Kumar Mishra, Shubha Shukla, Rakesh Maurya, Prabhat R. Mishra, Ritu Trivedi

**Affiliations:** 1 Division of Endocrinology, CSIR-Central Drug Research Institute, Lucknow, 226031 India; 2 Division of Medicinal and Process Chemistry, CSIR-Central Drug Research Institute, Lucknow, India; 3 Division of Pharmacology, CSIR-Central Drug Research Institute, Lucknow, India; 4 Division of Pharmaceutics, CSIR- Central Drug Research Institute, Lucknow, India; 5 Academy of Scientific & Innovative Research (AcSiR), CSIR-Central Drug Research Institute, Lucknow, 226031 India

**Keywords:** Cartilage, Monosodium iodoacetate (MIA), Osteoarthritis (OA), *Spinacia oleracea* extract (SOE)

## Abstract

**Background:**

*Spinacia oleracea* is an important dietary vegetable in India and throughout the world and has many beneficial effects. It is cultivated globally. However, its effect on osteoarthritis that mainly targets the cartilage cells remains unknown. In this study we aimed to evaluate the anti-osteoarthritic and chondro-protective effects of SOE on chemically induced osteoarthritis (OA).

**Methods:**

OA was induced by intra-patellar injection of monosodium iodoacetate (MIA) at the knee joint in rats. SOE was then given orally at 250 and 500 mg.kg^− 1^ day^− 1^ doses for 28 days to these rats. Anti-osteoarthritic potential of SOE was evaluated by micro-CT, mRNA and protein expression of pro-inflammatory and chondrogenic genes, clinically relevant biomarker’s and behavioural experiments.

**Results:**

In vitro cell free and cell based assays indicated that SOE acts as a strong anti-oxidant and an anti-inflammatory agent. Histological analysis of knee joints at the end of the experiment by safranin-o and toluidine blue staining established its protective effect. Radiological data corroborated the findings with improvement in the joint space and irregularity of the articular and atrophied femoral condyles and tibial plateau. Micro-CT analysis of sub-chondral bone indicated that SOE had the ability to mitigate OA effects by increasing bone volume to tissue volume (BV/TV) which resulted in decrease of trabecular pattern factor (Tb.Pf) by more than 200%. SOE stimulated chondrogenic marker gene expression with reduction in pro-inflammatory markers. Purified compounds isolated from SOE exhibited increased Sox-9 and Col-II protein expression in articular chondrocytes. Serum and urine analysis indicated that SOE had the potential to down-regulate glutathione S-transferase (GST) activity, clinical markers of osteoarthritis like cartilage oligometric matrix protein (COMP) and CTX-II. Overall, this led to a significant improvement in locomotion and balancing activity in rats as assessed by Open-field and Rota rod test.

**Conclusion:**

On the basis of in vitro and in vivo experiments performed with Spinacea oleracea extract we can deduce that SOE has the ability to alleviate the MIA induced deleterious effects.

**Electronic supplementary material:**

The online version of this article (10.1186/s12906-018-2117-9) contains supplementary material, which is available to authorized users.

## Background

Osteoarthritis (OA) is a high prevalence disease with socio-economic impact. It afflicts mainly the weight-bearing joints such as hips and knees, and causes physical disabilities. Over 100 million people worldwide suffer from OA [1]. It is a common rheumatologic problem with a prevalence of 22% to 39% in India [2]. Despite, the identified risk factors the exact pathogenesis of osteoarthritis remains unclear. As per reported literature, currently, there is no effective treatment that can cure osteoarthritis leaving joint replacement as the only therapeutic option in patients with severe osteoarthritis [3]. Articular cartilage degeneration is the primary concern in osteoarthritis. Cartilage degradation is associated with structural and metabolic changes in joints such as subchondral bone sclerosis and synovial membrane inflammation [4]. Biomechanical and biochemical interactions with subchondral bone and other joint tissues play important roles in maintaining homeostasis of articular cartilage [5]. Subchondral bone provides the mechanical support for the overlying articular cartilage during the movement of load-bearing joints and experience a constant adaptation in response to changes in the mechanical environment through modelling or remodelling [6]. Clinically, osteophyte formation is the characteristic marker of osteoarthritis [7]. Studies suggest that slow destructive process of joints results from the combination of mechanical stress, inflammation, and biochemical factors that includes mainly reactive oxygen species (ROS) and matrix metalloproteinases (MMPs) [8]. Prophylactic and symptomatic therapies are still available that reduce inflammation. However, therapies that can cure osteoarthritis are still far from reach.

The role of phyto-pharmaceuticals in the pathogenesis of osteoarthritis has drawn a lot of attention in recent years [9]. *Spinacia oleracea* which is cultivated globally is an important dietary food vegetable and a common raw material in the food processing industry [10]. The fresh leaves are cooked and taken as food. *Spinacia oleracea* has been used as dietary supplements or alternative medicinal foods in Indians as well as throughout the world. Spinach has been used as prevention or cure for diabetes [11], heart injury [12], neural disorders [13], hormone replacement therapy [14], anti-cancer [15] and many more. Currently, it is used as proper health supplement and clinician suggests as healthy food which fight against many disease conditions and health supplements. However, there are no reports concerning the osteoarthritis and chondro-protective effect of leaves of *Spinacia oleracea.* We have previously shown the osteogenic effects of *Spinacia oleracea* extract (SOE) in post-menopausal bone loss and fracture healing activity [16]. In drill hole fracture healing model, SOE not only stimulated osteoblast cells but also mobilized chondrocyte proliferation at the injury site [16]. This prompted us to investigate its role in subchondral bone pathology and articular cartilage degeneration during the progression of osteoarthritis. Studies report that injection of monosodium iodoacetate (MIA) into the knee joints of rats is considered a suitable model for osteoarthritis and used for evaluation of chondroprotective activity of novel agents which also resembles the phenomena observed in human OA [17]. Therefore, the present study was hypothesized to investigate the effect of SOE in cartilage degenerative disease at articular cartilage tissue region with an objective to search and develop novel therapeutic alternatives for the prevention and management of chondrocytes related disorders.

The present study is being carried out to investigate the effect of oral administration of SOE on pathological changes in MIA induced OA in in vivo settings in rats.

## Methods

### Reagents and chemicals

Cell culture media and supplements such as Dulbecco’s Modified Eagle Medium: Nutrient Mixture F-12 (DMEM/F-12) Media, Fetal bovine serum (FBS), antibiotic cocktail, 3-(4,5-dimethylthiazol-2-yl)-2,5-diphenyltetrazolium bromide (MTT), safranin-o and toluidine blue dye, 1, 1-diphenyl-2-picrylhydrazyl radical (DPPH), 2′-azinobis [3-ethylbenzthiazoline]-6-sulfonic acid (ABTS), and Dimethyl methylene blue (DMMB) were purchased from Sigma-Aldrich (St. Louis, MO, USA). Complementary DNA (cDNA) synthesis kit was purchased from Thermo Fisher Scientific (Waltham, Massachusetts, USA) and SYBR Green kit was obtained from Genetix (New Delhi, India). Rat CTX-II (ELISA KIT E-EL-R2554) and Rat COMP (ELISA kit E-EL-R0159) were purchased from Elabsciences (Elabsciences biotechnology co ltd, Hubei province, China).

### Preparations of Spinaciaoleracea extract (SOE)

The leaves of *Spinacia oleracea* were collected from district Lucknow, India during the month of November–December, by Dr. Rakesh Maurya and co-workers. It was identified and authenticated by Dr. Lal Babu Choudhary, Principal Scientist at Plant Diversity, Systematics & Herbarium Division CSIR-National Botanical Research Institute, Lucknow India. An institutional Code no. (CDRI-2492) had been deposited in herbarium of investor laboratory, Division of medicinal and process chemistry, CDRI, Lucknow for future reference.

The fresh *Spinacia oleracea* leaves (30 kg) were cut into small pieces and placed in glass percolator with ethanol (20 L) and allowed to stand at room temperature for approx.24 h. The percolate was collected. This process of extraction was repeated for four times. This extract was further filtered and concentrated under reduced pressure at 45 °C. The extract thus obtained was further dried in a vacuum desiccator to remove remaining ethanol and moisture and finally, 560 g (1.8% yield) weight of extract was obtained. This dried extract (100% of *Spinacia oleracea* leaves) was further used for different in-vitro and in-vivo studies as described previously by us [16].

### MTT (3-(4, 5-Dimethylthiazol-2-yl)-2, 5-diphenyltetrazolium bromide) assay

Cell viability was assessed using an MTT assay based on the ability of mitochondria of viable cells to convert soluble MTT into an insoluble purple formazan reaction product. First, dissolve SOE in 10 μl DMSO then it’s made up to 1.0 mg/ml by DMEM media; this is used as stock solution. This SOE stock is used for treatment on cultured rat articular chondrocytes cells (RAC) at different concentrations ranging from 1.95 to 1000 μg/ml.RAC was isolated from knee of young pups as per previously published protocols [18]. In brief, chondrocyte cells were isolated from distal femoral and proximal tibial condyles of 1 day-old Sprague-Dawley rats. Condyles tissue was isolated, cleaned from surrounding tissue and digested with trypsin for 30 min then processed in collagenase solution (2 mg/ml) at 37 °C for 4 h followed by passing through a 70 μm filter. Isolated cells were cultured for 48 h then trypsinized and 1 × 10^− 3^ cells/well were seeded in 48 wells plate. After 80–90% confluences of articular chondrocyte cells in monolayer culture, cells were treated with different concentrations of SOE for 24 h in DMEM/F12 medium with 10% fetal bovine serum [19]. After 24 h of SOE treatment, RAC cells were treated with MTT solution (5 mg/ml in Dulbecco’s modified Eagle’s medium (DMEM/F12) without phenol red; Sigma) for 2 h. The MTT solution was then aspirated and replaced with 200 ml/well dimethyl sulfoxide (DMSO). Detection was done at 540 nm OD by spectrophotometer [20].

### 2, 2-diphenyl-1-picryl-hydrazyl-hydrate (DPPH) antioxidant assay

The antioxidant activity of the SOE was measured in relationships of hydrogen donating ability using DPPH assay [21]. In this assay, SOE was added with DPPH that gave changes in spectrophotometric measurement when measured at 517 nm according to previously published protocol [21]. Ascorbic acid (AA) was used as positive control. Briefly, 0.1 mM solution of DPPH in methanol was prepared and SOE concentrations ranging from 1.95 to 1000 μg/ml were evaluated for antioxidant potential. The DPPH scavenging effect was measured as follows: DPPH scavenging effect (%) = ((Abc – Abs) / Abc) × 100. (Abc is value of DPPH without the sample; Abs is value of DPPH with SOE at 517 nm [22, 23].

### Lipid peroxidation inhibition activity

Malon-dialdehyde (MDA) assay was used to determine the lipid peroxidation inhibition effect of SOE as described by a previous study [24]. Briefly rat liver tissues (2.0 g) were sliced and homogenized in 10 ml 15 mM KCl–Tris-HCl buffer (pH 7.2). The reaction solution (0.25 ml liver homogenate, 0.1mlTris-HCl buffer (pH 7.2), 0.05 ml 1 mM ascorbic acid, 0.05 ml 4 mM FeCl2) and 0.5 ml of plant extract of different concentration of SOE were taken in a tube. The reaction tube was incubated at 37 °C for 1 h. After incubation, 0.5 ml 0.1 N HCl, 0.2 ml 9.8% sodium dodecyl sulphate, 0.9 ml distilled water and 2 mL 0.6% thio-barbituric acid (TBA) were added to each tube and vigorously shaken. Then, the tubes were placed in a boiling water bath at 95 °C for 30 min. After cooling, the flocculent precipitate was removed by adding 5 ml 푛-butanol, mixed well, and centrifuged at 2500 RPM for 10 min. The absorbance (ABS) of the supernatant was measured at 532 nm and percentage of lipid peroxidation by SOE was measured by following equation [25]. Lipid peroxidation inhibition (%) = [*A*control – *A*sample **/***A*control] × 100, where *A*_control_ is absorbance of control and *A*_sample_ is absorbance of sample (SOE extract).

### 2, 2′-azinobis [3-ethylbenzthiazoline]-6-sulfonic acid (ABTS) free radical scavenging assay

The antioxidant potential of the *Spinach oleracia* extracts (SOE) was measured using 2, 2′-azinobis [3-ethylbenzthiazoline]-6-sulfonic acid (ABTS) assay [22, 23]. In brief ABTS(14 mM), and potassium per sulphate (4.88 mM) was mixed, then left overnight (12~ 16 h) at room temperature in the dark. SOE concentration ranging 15.6 to 1000 μg/ml was measured by adding with ABTS solution to initiate the reaction. The absorbance was read at wavelength 415 nm with incubating for 15 min by a spectrophotometer (molecular devices, USA). The antioxidant capacity of each concentration of the extract was determined based on the reduction of ABTS absorption by calculating percentage of antioxidant activity as given in DPPH assay [26].

### Cartilage dissection and explant culture for DMMB assays

Articular cartilage tissues were obtained from knee joints of healthy SD rats [27]. Articular cartilage was disinfected by antibiotics and further washed with PBS before using for explant culture. Cartilage tissues were chopped and placed in DMEM+ F12 media for incubation for 24 h (37 °C, 5% CO2). After incubation, media was removed and replaced with following treatments: untreated culture media (control), IL-1β (10 ng/ml) and IL-1β with most effective doses of SOE as 500, 250, and 125 μg/ml. Explants were incubated (37 °C, 5% CO2) for 6 days and collected the supernatants for evaluation of proteoglycan release., The metachromatic dye 1,9 dimethyl methylene blue (DMMB) was used to quantify the amount of released proteoglycan especially sulphated glucosamineglycan’s (GAGs). DMMB activity was measured according to the previously developed protocol with little modification. On a 96-well plate, 1:1 sample and DMMB solution were added to each well, before reading the plate, it was kept in dark for 10 min then OD was measured by spectrophotometer at 540 nm. OD of DMMB is directly proportional to the release of proteoglycans [28].

### Western blot analysis

Rat articular chondrocyte were grown in complete media with and without treatment with 3-Methyl-6,7-(methylenedioxy) quercetagetin and 3-O-Methylpatuletin for 48 h. Cells were washed in chilled PBS and lysed by lysis buffer from Sigma Aldrich (St. Louis, MO, USA) containing a protease inhibitor mixture Sigma Aldrich (St. Louis, MO, USA). 30 μg of protein lysate were loaded in 12% polyacrylamide gel, followed by transfer on PVDF membrane. Membrane blocking was done by 5% BSA (bovine serum albumin) and then incubated with primary antibody at 4 °C overnight. Membrane were then washed and probed with horseradish peroxidase-conjugated secondary antibody (abcam®, Cambridge, USA) and visualized by Chemi-luminescence system (LS4000 GE Healthcare Life Sciences, India) [29].

### Induction of osteoarthritis (OA) and experimental design

The aim of this study was to assess the effect of SOE on MIA induced OA in the biological system of *Sprague-dawley* rats. For this experiment, 55 adult healthy and pathogen free female rats were used. Rats were divided into four groups (*n* = 10 in control group and *n* = 15 in each group of MIA induced rats. Adult healthy and pathogen free female *Sprague-dawley* rats, 14–16 weeks of age, (180 to 200 g) were obtained from the National Laboratory Animal Centre, Lucknow India. Animals were kept in a 12 h light-dark cycle, five animals in each steel cage with controlled temperature (22–24 °C), humidity (50–60%) and food and drinking water was supplied ad-libitum. This experiment was carried out in the Animal Centre of the central drug research institute for 28 days.

Rats were anesthetized by cocktail of Ketamine and Xylazin (9:1 ratio). After anesthetization all animals except control group received a single injection of MIA through the intra-articular joint of the left knee with 3 mg MIA in 50 μl saline [30]. The control group animals were injected with 50 μl of physiologic saline in their left knee [31]. All animals were closely observed every day and when primary symptoms like swelling in knee and walking disability was observed at the end of 3 days. These animals (*N* = 10, Total 30) were further selected for treatment of SOE and MIA control. SOE dispersed in 1.0% gum acacia in 250 or 500 mg.kg^− 1^ day^− 1^ doses and was given orally for 28 days once in a day. The control groups received only 1.0% gum acacia orally. After 28 days of treatment animals were euthanatized by CO_2_ exposure in the morning at laboratory.

### Histological analysis

Long bone tissues were isolated from differentially treated rats. Dissected tissues were cleaned by removing muscles and fixed in 4% formaldehyde. Tibia bone was decalcified in EDTA solution [32]. Sections of width 5.0 μm were cut by Leica RM2265 rotatory microtome with the help of sharp blade. Sections were stained with Safranin O and Touidine Blue. Photomicrographs of stained sections were captured using microscope (Evos XL, Life technologies).

### Micro computated tomography (micro-CT) at femoral condyles and tibia plateau region

The effects of SOE on micro-architectural deterioration in MIA induced osteoarthritis model were evaluated with micro computed tomography (Sky Scan 1076 scanner; Sky Scan, Aartselaar, Belgium). Femoral condyles and tibial plateau regions were scanned. Bones of all animals of each group were scanned at voxel size of 18 μm, at a voltage of 70 kV, a current of 142 mA, field of view of 35 mm, by using filter 1.0 mm aluminium plate, 0.8-degree rotation step with full width [33]. Total tissue volume (TV), trabecular bone volume (BV/TV, %), trabecular number (Tb.N, mm^− 1^), and trabecular separation (Tb.Sp, mm) and trabecular bone pattern factors (Tb.pf) (mm^− 1^), were calculated by BATMAN software provided with the μCT machine [34].

### Quantitative real-time polymerase chain reaction analysis

The bones were excised during autopsy, removed attached tissue on bone, cleaned and collected in RNA later. For RNA isolation, bones were cut and upper head part containing cartilage and subchondral region were taken for genes expression study. First, isolated bone tissues were grinded in liquid nitrogen, and total RNA was isolated by TRIzol according to the manufacturer’s protocol. Cdna was synthesized using a kit as per manufacturer’s protocol from 1 μg of RNA [35]. Forward and reverse primers were designed on the basis of previously published cDNA sequences using the Universal Probe Library for the all genes mentioned in Table 1. Quantitative real time PCR was performed by using SYBR green for determination of anti-osteoarthritis effect of SOE at gene expression level of different genes. β-actin was used as internal control [36].

**Table 1:** Primer sequences of various rat genes used for qPCR

Oligo name	Sequence (5’ to 3’)
*COL-1*	F: CAT.GTT.CAG.CTT.TGT.GGA.CCT R: GCA.GCT.GAC.TTC.AGG.GAT.GT
*IL1β*	F: TGT.GAT.GAA.AGA.CGG.CAC.AC R: CTT.CTT.CTT.TGG.GTA.TTG.TTT.GG
*Acan*	F: AAT.GGG.AGC.CAG.CCT.ACA.C R: TTG.AGA.GGC.AGA.GGG.ACT.TT
*SOX9*	F: TGA.AGA.AGG.AGA.GCG.AGG.AA R: CAT.AGC.CCT.TCA.GCA.CCT.G
*COL10a1*	F: CAC.AGC.CAT.TTC.GAG.CTT.TT R: TCT.AAG.TTG.CCC.CAG.GTA.CG
*MMP13*	F: TGG.ACA.AGC.AGC.TCC.AAA.G R: GTC.CAG.ACC.GAG.GGA.GTG
*MMP9*	F: CCT.CTG.CAT.GAA.GAC.GAC.ATA.A R: GGT.CAG.GTT.TAG.AGC.CAC.GA
*MMP1a*	F: GGT.GAT.ATT.GTG.TTC.GCC.TTC R: TCA.GGT.CCA.TCA.AAT.GGG.TTA
*TNF-α*	F: TGA.ACT.TCG.GGG.TGA.TCG R: GGG.CTT.GTC.ACT.CGA.GTT.TT
*TIMP-1*	F: CAG.CAA.AAG.GCC.TTC.GTA.AA R: TGG.CTG.AAC.AGG.GAA.ACA.CT
*COL2a1*	F: CCA.GGT.CCT.GCT.GGA.AAA R: CCT.CTT.TCT.CCG.GCC.TTT
*BMP-2*	F: CCC.CTA.TAT.GCT.CGA.CCT.GT R: AAA.GTT.CCT.CGA.TGG.CTT.CTT
*TIMP-2*	F: CGT.TTT.GCA.ATG.CAG.ACG.TA R: GAT.GGG.GTT.GCC.ATA.GAT.GT
*Β-ACTIN*	F: CCC.GCG.AGT.ACA.ACC.TTC.T R: CGT.CAT.CCA.TGG.CGA.ACT

*F* Forward Primer, *R* Reverse Primer

### Open-field activity test

The locomotor activity was examined on 28th day post- surgery in a sound proof room by Opto-Varimex-5 (Columbus Instruments, USA) which was consist of Plexi glass transparent cages (42.5 cm X 42.5 cm arena size) with a grid of infrared emitters and detectors in order to automatically detect activity measures [37].

### Rota rod test

Rats were individually placed on rotating rod (7.5 cm diameter) and trained through three different sessions on three consecutive days (one session/ day), whereby each session included four separate training trials. Rats were trained at 5 rpm on day 1, at 10 rpm on day 2 and at 15 rpm on day 3. At the end of training sessions, animals were tested to ensure that they could maintain themselves on rotating rod for 300 s. On 28th day post-surgery, each group of animals was subjected to Rota rod test to evaluate the latency to fall was recorded [37].

### GSTs (glutathione S-transferases) activity in serum

GST activity of control, MIA and different dose of SOE was measured in serum by glutathione S-transferase assay kit (Cayman USA, 703,302) according to manufacturer protocols. Briefly, 150 μl assay buffer provided with kit, 20 μl glutathione, 20 μl serum sample and finally add 10 μl 1-chloro-2, 4-dinitrobenzene (CDNB). This reaction mixture was carefully shacked and read at 340 nm by ELISA plate reader (molecular devices, USA) [38].

### Elisa

Urinary CTX-II (C-telopeptide of type II collagen) [39] and serum COMP (cartilage oligomeric matrix protein) [40], are biochemical markers for uses in progression of joint destruction. Urinary CTX-II and serum COMP concentrations were determined after 28 days treatment of SOE by enzyme-linked immunosorbent assay kits as per manufacturer’s protocols [41].

### Statistical analysis

All data is represented as Mean ± standard error of the mean (Mean ± S.E.M). Group differences were determined using one-way analysis of variance (ANOVA) with neuman–keuls post hoctests using Prism version 5.0 software. Probability values of *p <* 0.05 were considered to be statistically significant. Data for DPPH, ABTS, LPI, DMMB, MTT *n* = 4 and all assays were replicated three times. Data for qRT-PCR *n* = 3, serum/urine parameter *n* = 6 and micro CT analysis *n* = 10 samples were taken for analysis. Results were obtained from minimum three independent experiments in triplicate and are expressed as Mean ± S.E.M. For significance, *P < 0.05 was* used in each group, and results were reproducible and there were no disagreements amongst the blinded assessors (RT, PRM and RM).

## Results

### Cell viability assay of SOE

**Fig. 1:**
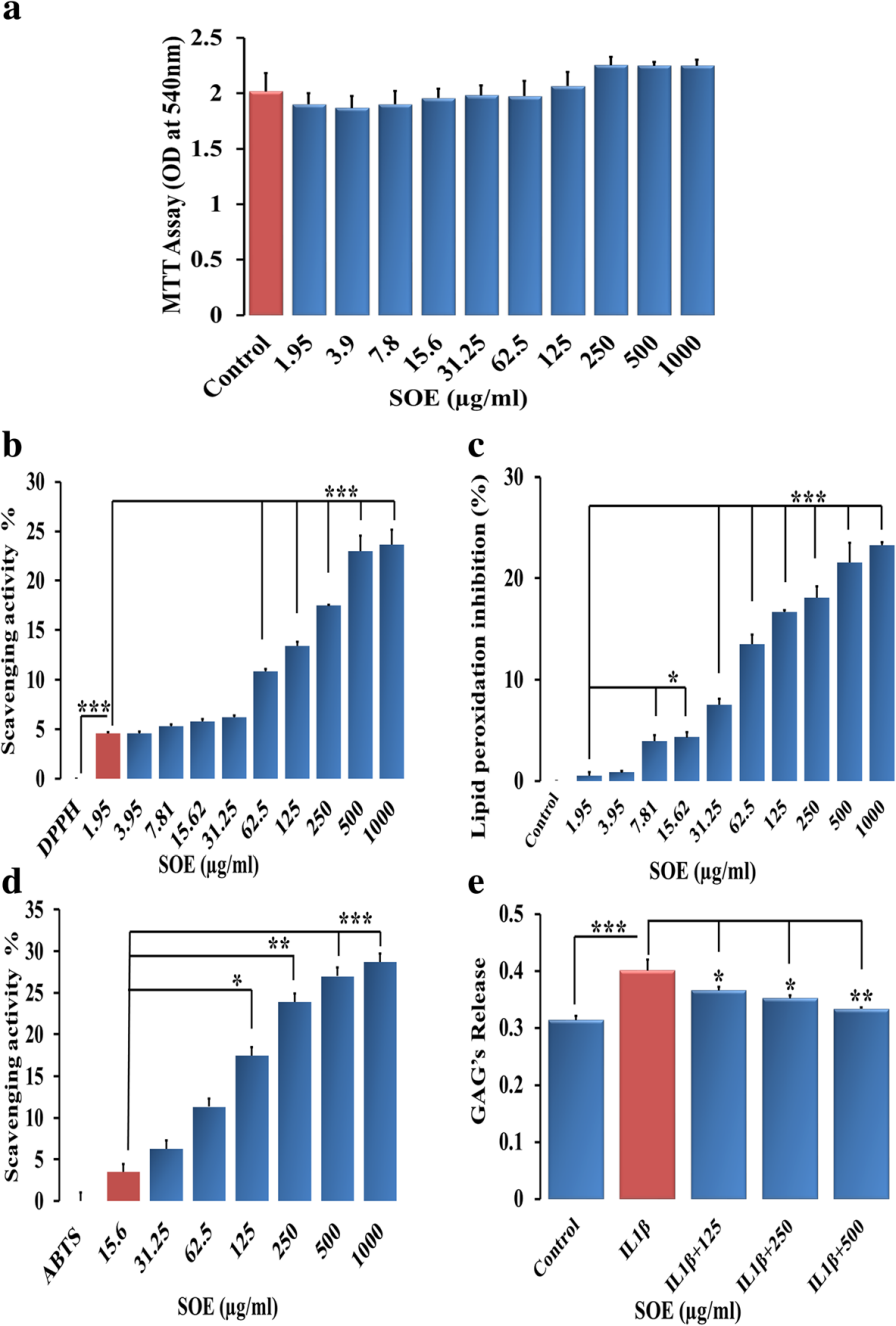
SOE has antioxidant activity and is non-toxic for chondrocytes. **a** MTT assay at different concentrations of SOE (μg/ml) showed that it is safe and does not impart toxic effects on chondrocyte viability. **b** Anti-oxidant ability of SOE by DPPH and (**c**) lipid peroxidation inhibition by MDA assay (D) Free radicals scavenging activity by ABTS assays and (E) GAG release measurement by DMMB assays. All values are expressed as mean ± S.E.M. (*n* = 6); *P < 0.05; **P < 0.01, ***P < 0.001 compared to control

Cell viability after treatment with different concentrations of SOE was determined by MTT assay on rat articular chondrocytes cells. Data in (Fig. 1a) shows that the SOE concentration from 1.95 to 1000 μg/ml was safe for use and did not alter cells viability at any dose examined in this study. In addition, we evaluated SOE efficacy in accute toxicity rat model as shown in HE statining (see Additional file 1: Figure S1). We also assesed biochemical parameters ALT and AST as liver health indicators (see Additional file 2: Figure S2) and its 'materials and method' is given in text file (Additional file 3).

### Free radical scavenging activity of SOE

We next assessed the antioxidant activity of SOE at different doses using the DPPH free radical scavenging method. SOE at concentrations between 1.95 to 1000 μg ml^− 1^ showed significant scavenging activity. Maximum scavenging activity by SOE was 17.52%, 23.02% and 23.65% at doses of 250, 500 and 1000 μg.ml^− 1^ respectively (Fig. 1b). Ascorbic acid was taken as positive control as shown in Additional file: 4: Figure S3.

### Ex-vivo lipid peroxidation inhibition activity of SOE

Lipid peroxidation inhibition activity was measured ex-vivo by determining the malondialdehyde (MDA) in rat liver homogenate. Inhibition of lipid peroxidation indicated anti-oxidant property of plant extracts. Experiment showed that SOE exhibits MDA inhibition at 250, 500 and 1000 μg.ml^− 1^ by 18.05%, 21.53% and 23.28% respectively (Fig. 1c).

### Free radical cation scavenging activity of SOE

ABTS radical scavenging method was adopted for the determination of antiradical and antioxidant activity of SOE and the obtained results are showed in Fig. 1d. We checked anti-oxidant potential of SOE for the concentration range from15.6 to 1000 μg ml^− 1^. We observed maximum anti-oxidant potential of SOE at 250, 500 and 1000 μg.ml^− 1^that was 24.23%, 27.37% and 28.95% respectively (Fig. 1d).

### Effect of SOE on GAG release

To assess the preventive effect of SOE we next assessed the glycosamine glycan (GAG) release in conditioned media of IL-1β treated rat articular chondrocytes as IL-1β activates MMPs that cleave the extracellular matrix components of the cartilage. After 6 days of culture in DMEM, we observed 27.75% increase of GAG release in media in the presence of IL-1β as compared to the control. Experiments in the presence of SOE (IL-1β + SOE) at concentration 125 μg.ml^− 1^, IL-1β + SOE at concentration250 μg.ml^− 1^ and IL-1β + SOE at concentration500 μg.ml^− 1^ inhibited GAG release by 8.34%, 11.84% and 16.38% respectively. This experiment suggests that SOE has the ability to prevent the destruction of the cartilage as caused by IL-1β-stimulation and thus GAG release in the media (Fig. 1e).

### Active components of SOE increase chondrogenic proteins

**Fig. 2:**
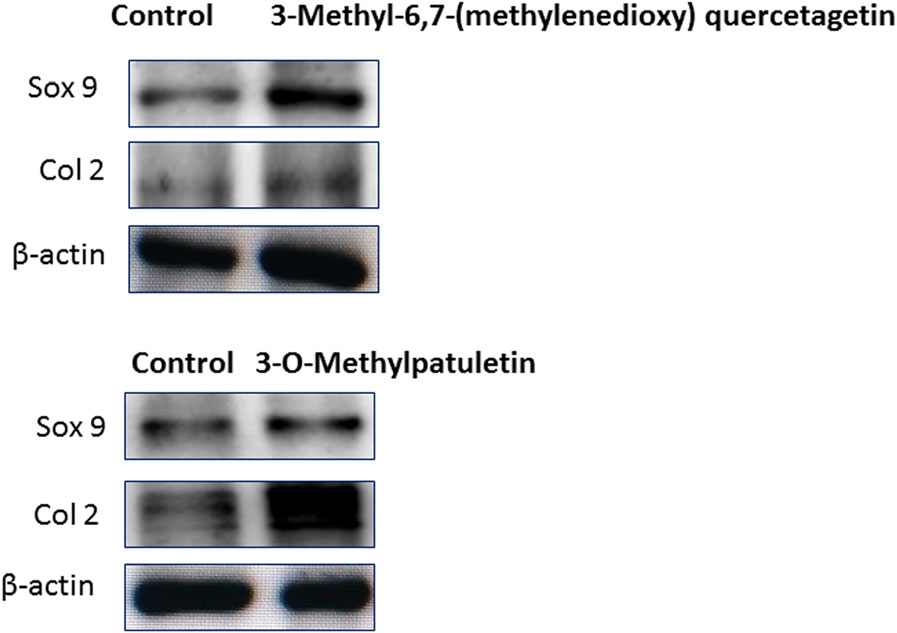
Effect of SOE on chondrogenic proteins. Western blot analysis of Sox 9 and Col 2 after 3-Methyl-6,7-(methylenedioxy) quercetagetin, and 3-O-Methylpatuletin (active compounds extracted from SOE) is represented after treatment in rat articular chondrocytes isolated from 5 days old pups

Treatment with 3-Methyl-6, 7-(methylenedioxy) quercetagetin and 3-O-Methylpatuletin two active compounds isolated from SOE increased protein expression of Sox 9 and Col-2 the chondroprotective markers in articular chondrocytes (Fig. 2) suggesting that the prevention of cartilage damage as observed by SOE administration may have been the contribution of these pure compounds. HPLC data of SOE  and its active components are exhibited in Additional file 5: Figure S4.

### Effect of SOE on articular cartilage and subchondral bone

**Fig. 3:**
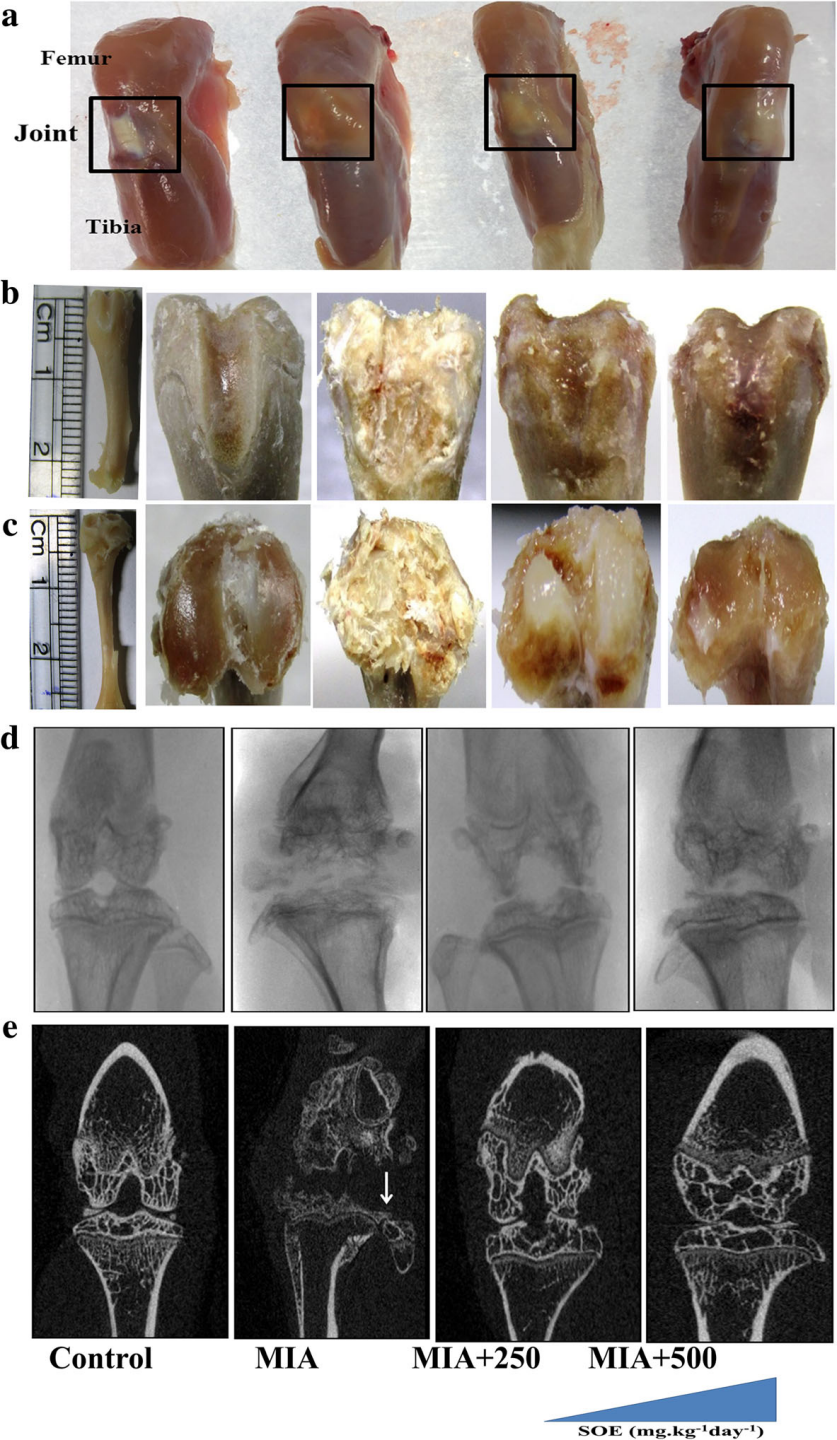
Effect of SOE treatment at articular and sub-chondral bone. **a** Images of excised knee from various groups showed morphological changes in different treated groups. **b** and **c** macroscopic photographs of femur and tibia respectively after termination of study. **d** Radiographic images of various groups and (**e**) 2D micro-CT images of knees. There was significant cartilage loss and sub-chondral bone parts in MIA-injected group (marked with an arrow). Treatment with SOE ameliorates articular degradation

Figure 3a represents the picture of gross morphological changes at the knee joint after 4 weeks of injection of MIA into the left knee. Grossly, the normal joints revealed smooth and shiny articular surfaces. The knees of the MIA-injected group showed irregular abrasions at the articular cartilage surfaces of the femoral condyle and the tibial plateau. Figure 3 represents the macroscopic photographs of the damaged articular cartilage of the femur condyles (B) and tibial plateau (C) after injection of MIA. OA was characterized by progressive cartilage destruction and alterations in subchondral bone structure. Figure 3d represents radiographies of the left knee joints. Radiographs of the control group showed normal state of the knee characterized with smooth articular surfaces. Due to MIA intra articular injection, joint represents with complete loss of joint space, irregularity in articular surfaces and atrophied femoral condyles and tibial plateau. Interestingly, the treatments with 250 and 500 mg.kg^− 1^ day^− 1^doses of SOE for four weeks restored the cartilage morphology. Further, after 4 weeks of MIA injection, μ-CT analysis revealed a significant loss in cartilage and subchondral bone parts in MIA group (Fig. 3e). In rat model of OA, the micro-CT analyses showed that treatment with 250 and 500 mg.kg^− 1^ day^− 1^of SOE suppressed osteoarthritis progression.

**Fig. 4:**
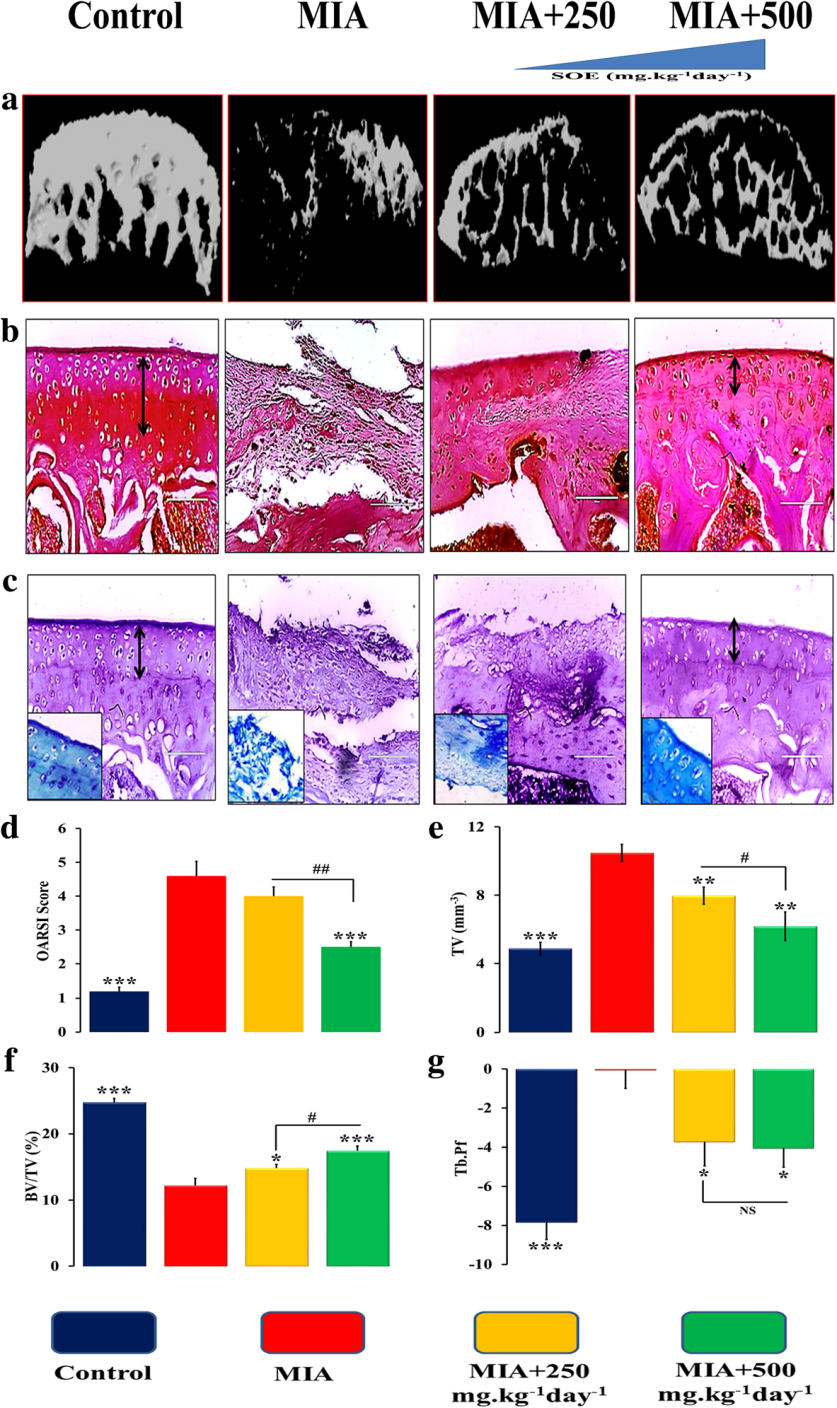
Histological and dynamic histological analysis of articular cartilage and subchondral bone in experimental groups. **a** 3D μ-CT images of articular cartilage. **b** safranin-O staining (**c**) toluidine blue staining of sagittal sections of tibia. Double headed arrow showing hyaline cartilage and calcified cartilage (**d**) OARSI score from five different sections *n* = 3 per group. Micro-CT analysis of sub-chondral bone (**e**) total tissue volume (TV), (**f**) bone volume/tissue volume (BV/TV and (**g**) trabecular porosity (Tb. Pf). All values are expressed as Mean ± S.E.M. (*n* = 6/group); *P < 0.05, **P < 0.01, ***P < 0.001 compared to the MIA group ^#^*P* < 0.05, ^##^*P* < 0.01, compared to the 250 mg.kg^− 1^ day^− 1^ dose, NS= non significant

Further, detailed analysis of tibial bone by micro computed tomography (μ-CT) indicated that MIA degrades cartilage substantially relative to sham-operated group (Fig. 4a). Histological analysis by safranin O (Fig. 4b) and toluidine blue (Fig. 4c) showed that MIA injected animals undergo maximum loss at articular region as compared to saline treated control animals with notable proteoglycan loss and disappearance of chondrocytes at the deep zone of articular cartilage. Figure 3b and Fig. 3c reflect intact articular cartilage in control group with SOE reducing the MIA-induced cartilage degradation. We observed that the dose of 250 mg.kg^− 1^ day^− 1^ of SOE had less effect on the structural changes in the joints as compared to the 500 mg.kg^− 1^ day^− 1^ dose. The 500 mg.kg^− 1^ day^−1^dose resulted in restoration of hyaline cartilage which is revealed by histology of articular cartilage.

Osteoarthritis Research Society International (OARSI) scores revealed degeneration of articular cartilage that was induced after MIA injection and progressed gradually (Fig. 4d).

OA is primarily associated with damage of articular cartilage that leads to increased bone remodelling followed by subchondral bone deterioration with the progression of the disease. Results of μ-CT at the subchondral bone site show that MIA induction increases tissue volume (TV) by 160% and decreased subchondral bone volume/tissue volume (BV/TV) ratio by 58% as compared to sham-operated controls. Increase in subchondral TV after MIA induced OA ultimately led to the reduction of the total BV/TV. Moreover, MIA disrupts the connectivity and microarchitecture of subchondral bone which was indicated by a significantly higher trabecular pattern factor (Tb.Pf) (Fig. 4e-g). Treatment of SOE at 500 mg.kg^− 1^.day^− 1^treatments decreased the TV by 48% and increased the BV/TV by 79% that resulted in a significant decrease in Tb.Pf by more than 200% after SOE administration. We also assessed μ-CT parameters at femoral condyles (see Additional file 6: Figure S5).

**Fig. 5:**
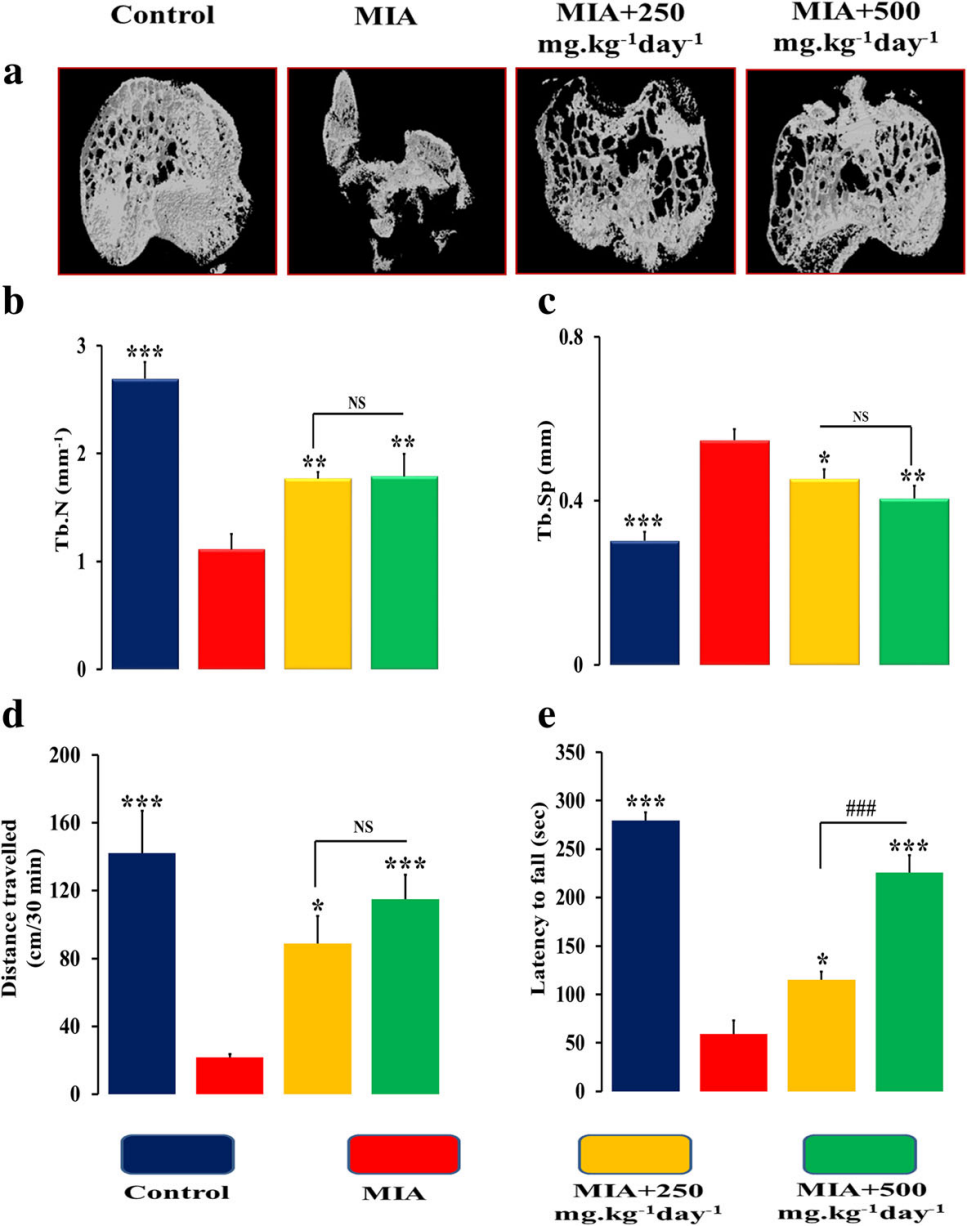
SOE improves micro-architectural and behavioural parameters. **a** Representative 3D μCT images of tibia plateau region of different groups. **b** Trabecular number (Tb.N, 1/mm); (**c**) Trabecular Separation; (Tb.Sp, mm) (**d**) Open-field activity and (**e**) Rota rod test to examine the locomotor function and motor coordination. All values are expressed as Mean ± S.E.M. (*n* = 6/group); *P < 0.05, **P < 0.01, ***P < 0.001 compared to the MIA group, ^###^*P* < 0.001 compared to the 250 mg.kg^− 1^ day^− 1^ dose, NS= non significant

We further assessed the trabecular bone by 3D- μ-CT that clearly showed that SOE has potentially lessened the harmful effects of MIA as indicated in the representative images Fig. 5a**.** MIA significantly decreased number of trabecules in bone as assessed by Tb.N (Fig. 5b) that leads to disruption of the trabecular connectivity and higher trabecular separation (Tb.Sp) in the MIA rats (Fig. 5c).The SOE dose of 250 mg.kg^− 1^ day^− 1^ reversed the MIA induced effects with increased Tb.N by ~ 58% (Fig. 5b) and decreased, Tb.Sp by ~ 17% (Fig. 5c) as compared to MIA group. On the other hand, the higher dose of 500 mg.kg^− 1^ day^− 1^ maintained the articular cartilage by increasing Tb.N by ~ 60% and decreasing Tb.Sp by ~ 26% compared to MIA group. Inter-dose comparisons show that SOE at 500 mg.kg^− 1^ day^− 1^was more effective in rescuing articular and sub-chondral bone loss.

### Open-field activity test and Rota rod test after SOE administration

The protective effect of SOE on the cartilage as observed in the above experiments was concluded on the basis of the behavioural experiments of locomotion. We observed that MIA treated rats significantly travelled for shorter distance in open-field area experiment (Fig. 5d) and decreased latency to fall as seen in Rota rod test (Fig. 5e), as compared to control group. This could have been because of the articular joint inflammation and destruction as observed by us or due to osteoarthritic pain. Observations showed that 500 mg.kg^− 1^ day^− 1^ SOE displayed significant improvement in distance travelled and increased in latency to fall in Open-field and Rota rod test respectively, when compared with MIA group. Although we observed positive changes with the lower dose (Fig. 5d and e) of SOE but the overall effect was more pronounced with the 500 mg.kg^− 1^ day-1 dose.

### Effects of SOE on the expression of pro-inflammatory cytokines and chondrogenic genes in articular cartilage region of knee joints

**Fig. 6:**
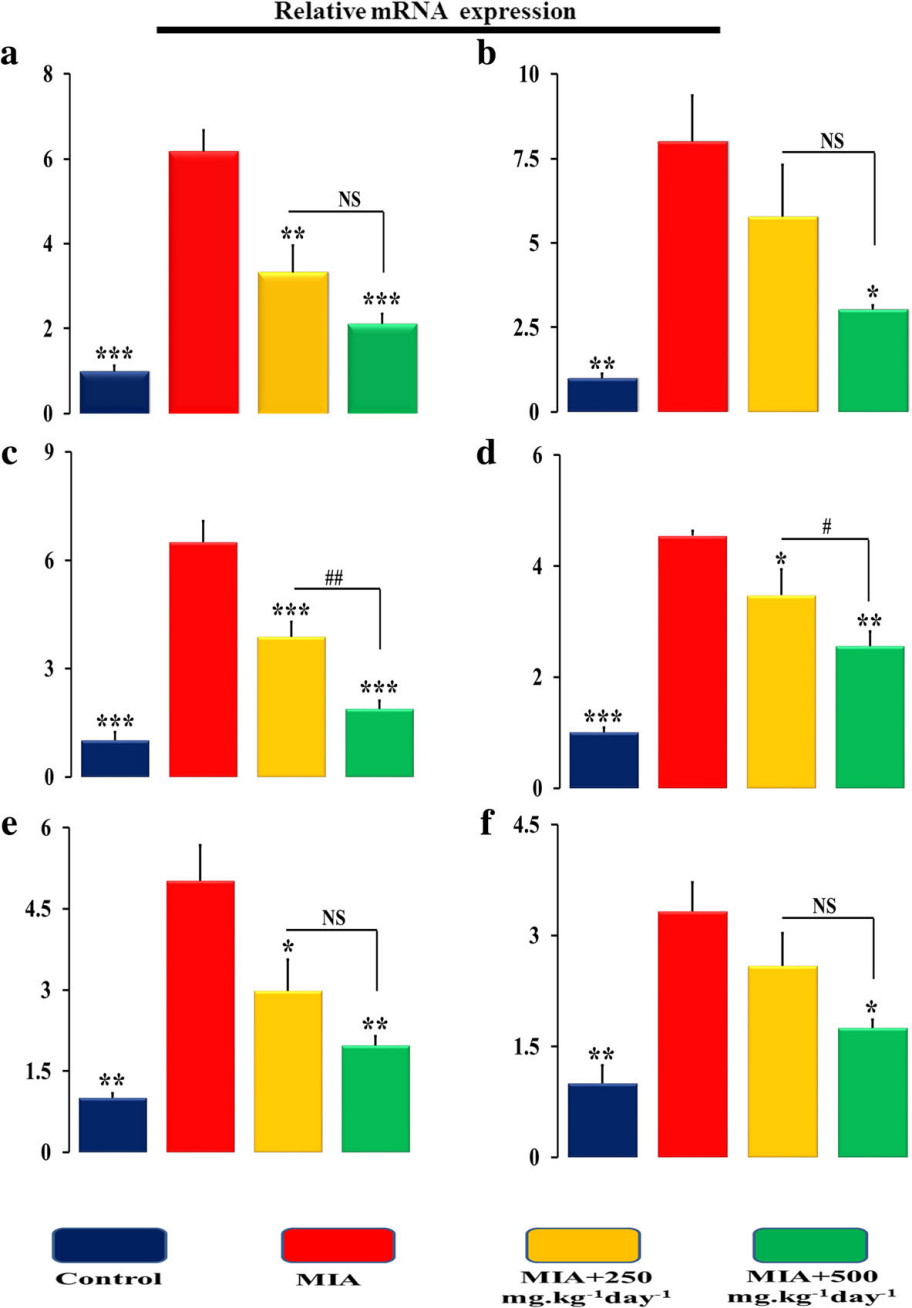
SOE inhibits MIA induced pro-inflammatory gene expression. qPCR determination of mRNA levels of pro-inflammatory genes (**a**) IL1-β, (B) TNF-α, (**c**) Col-10, (**d**) MMP-1, (**e**) MMP-3 and (F) MMP-13 in isolated bone region which contain cartilage and sub-chondral bone. All values are expressed as Mean ± S.E.M. (*n* = 6/group); *P < 0.05, **P < 0.01, ***P < 0.001 compared to the MIA group ^#^*P* < 0.05, ^##^*P* < 0.01, compared to the 250 mg.kg^− 1^ day^− 1^ dose. Interleukin-1 beta (IL-1β), Tumor necrosis factor alpha (TNFα), Collagen type 10 (Col10), Matrix metalloproteinases (MMPs) like interstitial collagenase (MMP-1), stromelysin-1 (MMP-3), and collagenase 3 (MMP-13), NS= non significant

**Fig. 7:**
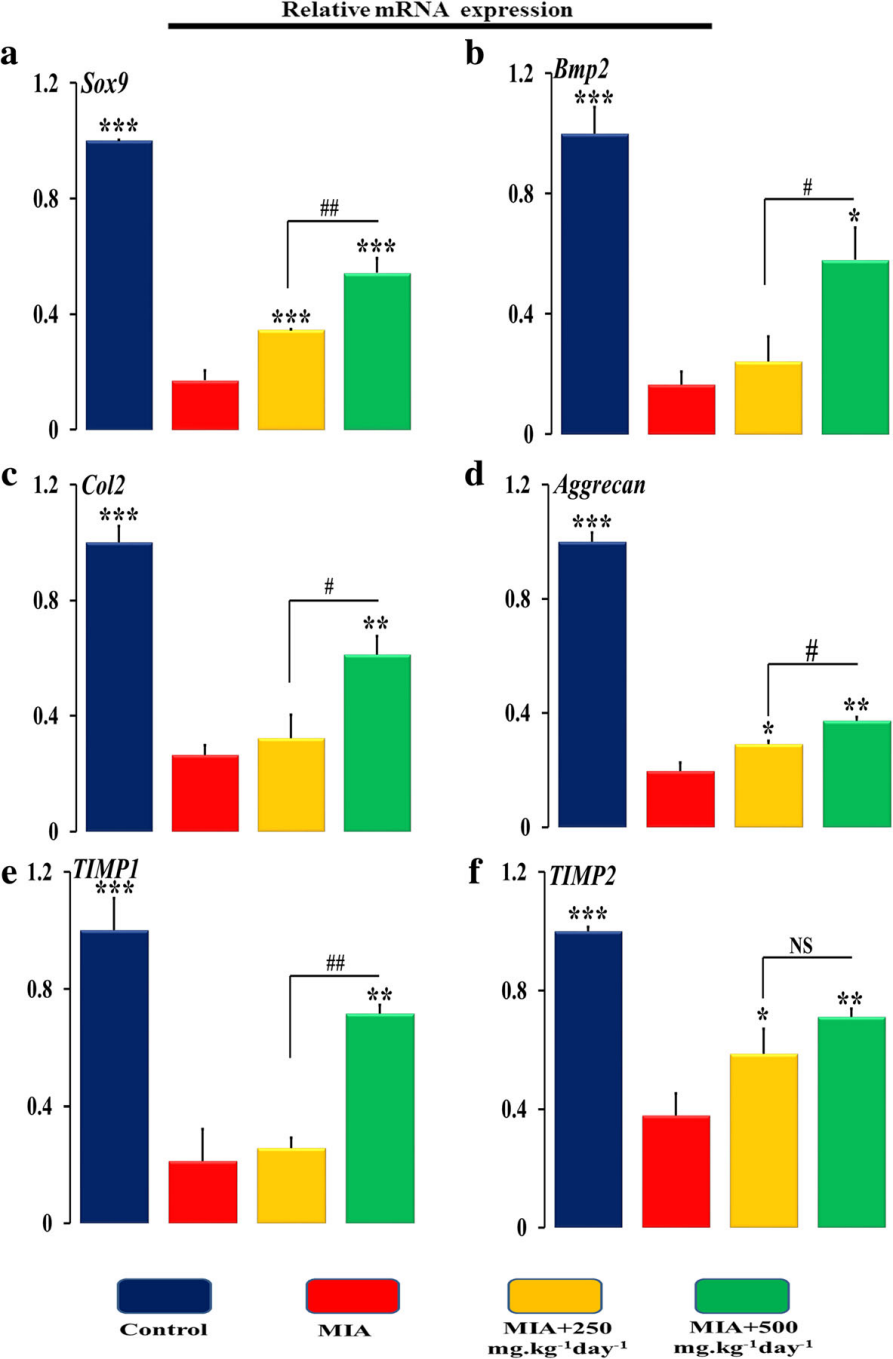
SOE increases MIA inhibited chondrogenic marker gene expression. qPCR determination of mRNA levels of chondrogenic genes (**a**) Sox9, (**b**) Bmp2, (**c**) Col2a, (**d**) Aggrecan, (**e**) TIMP-1 and (**f**) TIMP-2 in isolated bone region which contain cartilage and sub-chondral bone. All values are expressed as Mean ± S.E.M. (*n* = 6/group); *P < 0.05, **P < 0.01, ***P < 0.001 compared to the MIA group ^#^*P* < 0.05, ^##^*P* < 0.01, compared to the 250 mg.kg^− 1^ day^− 1^ dose. Sex determining region Y-box 9 (SOX9) (Fig. 6a), bone morphogenetic protein 2 (BMP2) (Fig. 6b), collagen type-II (Col2) Tissue inhibitors of matrix metalloproteinases (TIMPs), NS= non significant

At molecular level expression of pro-inflammatory and chondrogenic genes in isolated cartilage from the knee region of control, MIA and SOE administered animals were investigated. The progressive destruction of articular cartilage is caused by MIA injection that significantly upregulated the expression of pro-inflammatory genes interleukin-1 beta (IL-1β), tumor necrosis factor alpha (TNFα), and matrix-degrading enzymes (Fig. 6a-f). IL-1β and TNFα are considered as the major cytokines, involved in the onset and pathogenesis of OA. MIA increased expression of IL-1β by ~ 6.0 folds (Fig. 6a) and TNFα by ~ 7.0 folds (Fig. 6b). This led to an increase in expression of collagen 10 (COL10) (Fig. 6c) that increases hypertrophy like environment in the articular cartilage region. These cytokines further damaged the joints by enhancing the expression of a group of metalloproteinases like interstitial collagenase (MMP-1), stromelysin-1 (MMP-3), and collagenase 3 (MMP-13) (Fig. 6d-f). In addition to these harmful effects, MIA blocked chondrogenic activity by down regulating the synthesis of ECM components, like sex determining region Y-box 9 (SOX9) (Fig. 7a), bone morphogenetic protein 2 (BMP2) (Fig. 7b), collagen type-II (Col2) (Fig. 7c), and aggrecan (Fig. 7d). Further, tissue inhibitors of matrix metalloproteinases (TIMPs) are recognized as endogenous protease inhibitors of MMPs. MIA decreased the expression of TIMP1 (Fig. 7e) and TIMP2 (Fig. 7f).

When we administered SOE at 250 mgkg^− 1^ day^− 1^ and at 500 mg.kg^− 1^ day^− 1^ we observed reversal of the deleterious effects of MIA. The dose of SOE at 250 mgkg^− 1^ day^− 1^ significantly decreased the expression of IL-1β by ~ 46%, COL10 by~ 40%, MMP1 by ~ 22% and MMP3 by ~ 40%, and moreover, upregulated the expression of SOX9 by ~ 103%, aggrecan by ~ 52%, and TIMP2 by ~ 56% as compared to MIA group. When a higher dose of SOE was used we observed an inhibition by ~ 50% of the expression of inflammatory cytokines like IL-1β, TNFα, COL10, MMPs and restoration of the cartilage by upregulation of the extra-cellular matrix components by more than 100% in SOX9, BMP2, COL2, AGGRECAN, TIMP1, TIMP2. Inter-comparison between results associated with two different doses of 250 and 500 mg.kg^− 1^ day^− 1^ of SOE, we observed that the 500 mg.kg^− 1^ day^− 1^dose was more effective for restoration of the cartilage as a therapeutic treatment.

### Glutathione S-transferases (GSTs) activity in serum

**Fig. 8:**
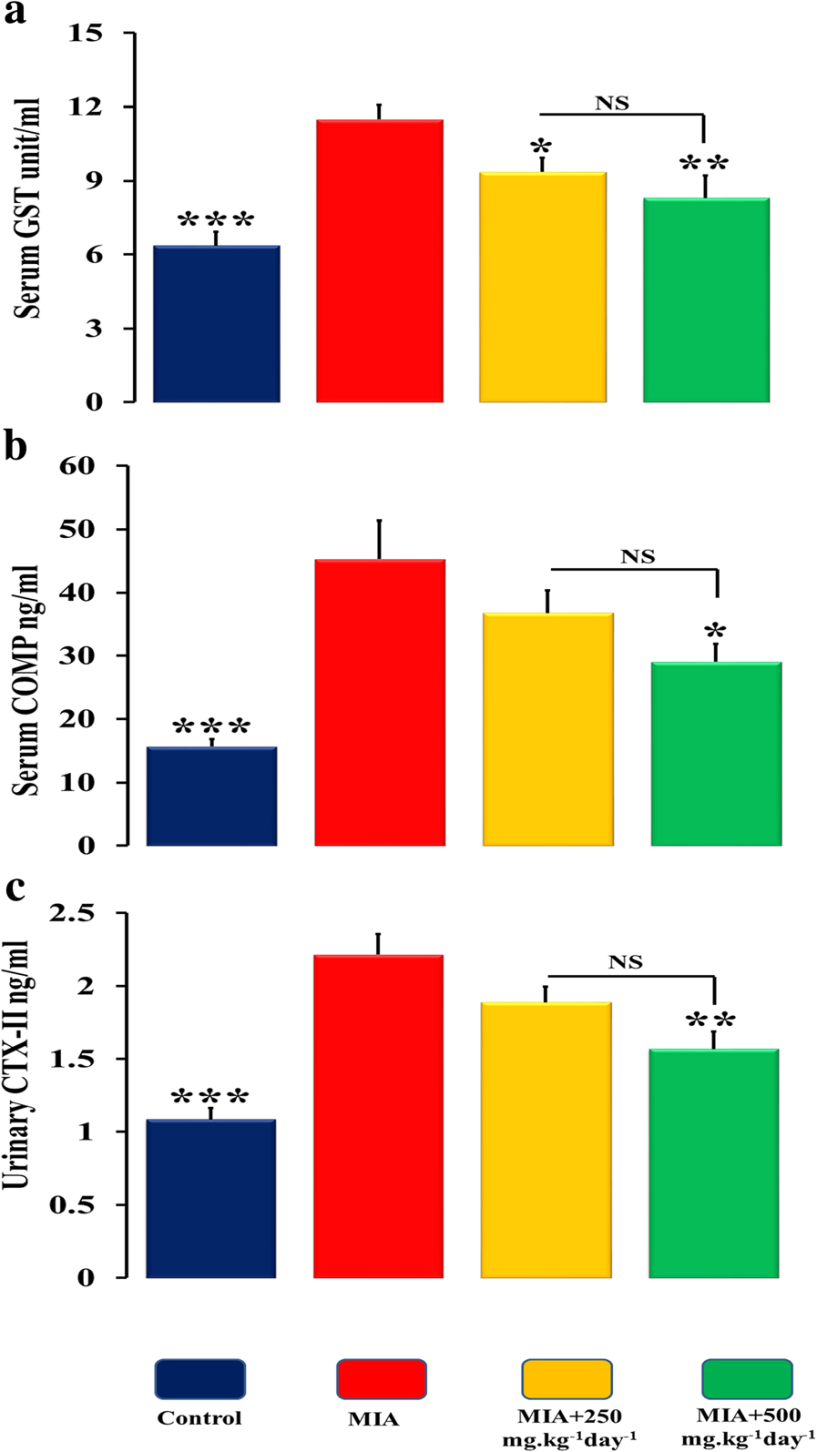
Effect of SOE on serum and urinary osteoarthritic markers. **a** Serum GST level measurements **b** serum COMP measurements and **c** urinary CTX-II levels. All values are expressed as Mean ± S.E.M. (*n* = 6/group); *P < 0.05, **P < 0.01, ***P < 0.001 compared to the MIA group compared to the 250 mg.kg^− 1^ day^− 1^ dose. GSTs (Glutathione S-transferases), serum COMP (cartilage oligomeric matrix protein) and Urinary CTX-II (C-telopeptide of type II collagen), NS= non significant

An increased serum GST pool of ~ 20% was found in serum of MIA injected group which indicated that MIA animals produced more GST activity to detoxify itself in response to the harmful effects of MIA. Oral administration of SOE decreased the GST activity by ~ 34% at 250 mg.kg^− 1^ day^− 1^and by ~ 30% at 500 mg.kg^− 1^ day^− 1^with respect to MIA group. No significance was observed within the SOE administered groups (Fig. 8a).

### SOE treatment decreases cartilage turnover marker in biological fluids

MIA increased the levels of serum COMP and urinary CTX-II, which are associated with progression of OA. SOE at 250 and 500 mg.kg^− 1^ day^− 1^ administration to MIA injected animals lowered the CTX-II and COMP levels as compared to MIA group however significant changes were observed at 500 mg.kg^− 1^ day^− 1^(Fig. 8b, c). The clinical markers highlight the potential of SOE in preventing loss of articular cartilage and subchondral bone as potential anti-osteoarthritic agent.

## Discussion

In this study, SOE was evaluated at physiological doses for its effect on MIA induced osteoarthritis in rodents. Chondrocytes are the only component cells that are capable of controlling vital activities of the articular cartilage [42]. Therefore, the saving of plausible number of cartilage cells in the joint’s articular structure of SOE treated group was not only interesting but also served as an important finding in OA pathogenesis and progression. Previous studies reported that inflammatory cytokines, chemokines, and other inflammatory markers are found higher in osteoarthritis patients [43].

Till date, complete cure for OA remains elusive and the management of OA is largely palliative focusing on the alleviation of symptoms only. Current recommendations for the management of OA include a combination of pharmacological (NSAIDs, DMOADs, paracetamol, etc.) and non-pharmacological interventions (weight loss and exercise). Pharmacological drugs include non-steroidal anti-inflammatory drugs (NSAIDs) that have analgesic and antipyretic actions, with risk of an upper gastrointestinal (GI) event like ulcer, perforations, bleeding and obstruction. Disease modifying OA drugs (DMOADs) are currently in clinical trial phase (like matrix metalloproteinase inhibitors, Fibroblast growth factor 18 (FGF-18), Interleukin 1 inhibitors, Inducible nitric oxide synthase) and long term safety of these drugs is still unclear [44].

Currently, use of herbal products for medicine and dietary supplements or as complementary and alternative medicine have become an important area of research in bone health especially orthopaedics, arthritis, rheumatic diseases, and musculoskeletal [45]. Herbal products have lot of therapeutics and dietary uses with low risk of side effects and toxicity [46–51]. Therefore, in current study, efforts are being made to elucidate the role of natural product like SOE for the prevention and cure of OA. In this regard, this study first evaluated the anti-oxidant and anti-inflammatory effects of SOE. ABTS and DPPH assays indicates that SOE have anti-oxidant and free radicle scavenging activity without using animals showing as a marker of preliminary identification of characterization of novel agent for treatment of OA.

Results taken together by DPPH, ABTS, and LPI experimental data revealed the antioxidant nature of SOE. Furthermore, when cartilage tissue was exposed to IL-1β, it underwent degradation and consequently released GAG, which is a typical clinical symptom of OA [52]. While treating with SOE, the released GAG was significantly reduced as measured by DMMB assay and showed anti-inflammatory potential of SOE.

Further, anti-osteoarthritic activity of SOE was studied under *in- vivo* conditions by making MIA induced osteoarthritic rat model, which resembles the phenomena observed in human OA of knee region and by increasing disease conditions like free radical generation and inflammation [17]. The MIA used in the present study to induced OA condition as MIA suppression of glyceraldehyde-3-phosphate dehydrogenase (GAPDH) that results in a reduction of glycolysis activity in the cartilage chondrocytes which further cause pathological structural/morphologic changes in articular cartilage. The MIA caused histopathological changes in articular cartilage structure which was confirmed by safranin-o and toluidine blue staining. Histological and ORASI data indicated that SOE treatment prevents the degradation of chondrocytes and extracellular matrix (ECM) components in articular region. Previous studies support that both the cartilage and subchondral bone communicate to each other through biomechanical and biochemical signalling pathways for maintaining homeostasis of the joint environment. Moreover, defects in articular cartilage increase various inflammatory cytokines and decrease the chondrocytes synthesis that further leads to increased bone remodelling and subchondral bone deterioration. Radiography, confirmed the above gross findings of SOE that were corroborated and confirmed quantitatively with micro-CT. It was found that 4 weeks post-treatment with SOE sustained the changes found especially in the joint spaces. These changes with SOE were brought about by improving the cartilage by increasing total bone volume to tissue volume and trabecular number that provide structural and functional strength to the cartilage. Whereas, in case of those treated with MIA, radiographs showed increased inter condylar fossa, severe joint space loss, atrophied femoral condyles and lack of articular surface smoothness. Impairment of joint space is an important feature in diagnosis and assessment of OA. MIA significantly decreased duration of rotation and balancing on moving rod that are related to inflammation swelling, pain and stiffness in joint whereas, SOE treatment showed increased joint mobility with increased travelling distance and decreased chance of latency to fall, suggesting its role in lessening pain and its associated symptoms.

Pathophysiology of OA, is also associated with increased inflammatory cytokines associated with the disruption of homeostasis of ECM component in OA as it is often subjected to target high mechanical load tissues such as joints by disturbing the catabolic and anabolic processes [53]. The cytokines like IL-1β and TNFα produced by the chondrocytes after defect in articular cartilage, especially IL-1β, play a significant role in the further degradation of cartilage. IL-1β and TNFα are the main cytokine instigators of cartilage degeneration in arthritis, and these induce MMP in chondrocyte cells [54]. Molecular analysis by qRT-PCR revealed that imbalance between the cartilage anabolic and inflammation related genes might account for the advanced condylar destruction and chondrocytic apoptosis following MIA induction that is balanced by SOE. MIA increased the size of articular chondrocytes by an increase in expression of Col10 that leads to upregulated osteogenic environment in the articular region and leads to progression of OA by subchondral bone deterioration. SOE treatment maintained chondrogenic environment of articular chondrocytes by decreasing the expression of Col10 in the joints. As shown by data, MIA affected the joints by enhancing the expression of a group of metalloproteinases, which have a destructive effect on cartilage. These metalloproteinases block chondrocyte activities by down regulating the synthesis of ECM components, interfering with the synthesis of the key structural proteins such as collagen type-II, Sox9 and aggrecan [55]. This was corroborated by the treatment of isolated pure compounds from SOE to rat articular chondrocytes suggesting that the active components present in SOE may be contributing to prevention from the damaging effect of MIA. For better understanding, the schematic diagram is shown (see Additional file 7: Figure S6) in which SOE exhibits chondro-protective effects on subchondral bone and causes the shifting of chondrocytes and cartilage homeostasis towards anabolism.

In addition, SOE treatment remarkably reduced the elevated serum level of GST, COMP and urinary CTX-II associated with MIA in a dose-dependent manner. These findings indicated that SOE treatment decreases the MIA induced inflammation, articular and sub-chondral bone loss. This study indicates that SOE has anti-osteoarthritic and chondro-protective effects in chemically induced osteoarthritis. However, confirmation of the effect of SOE on OA as observed by us further requires validation in higher animals and primates for extrapolation of data to humans.

## Conclusions

On the basis of in vitro and in vivo experiments performed with *Spinacea oleracea* extract and it’s isolated compounds we can deduce that SOE has the ability to alleviate MIA induced deleterious effects.

## Additional files

Additional file 1: **Figure S1.** H&E stained organ sections, isolated from rat after treatment of SOE (acute toxicity study). No noticeable abnormality was observed in major organs including kidney, liver, and spleen. (TIFF 9629 kb)

Additional file 2: **Figure S2.** Serum ALT and AST level in different groups after 28 days of treatment of SOE. No significant differences in control, MIA, 250 mg/kg, 500 mg/kg. All values are expressed as Mean ± S.E.M (*n* = 4/group). (TIFF 7976 kb)

Additional file 3: Supplementary information. (DOCX 16.5 kb)

Additional file 4: **Figure S3.** Ascorbic acid was used as positive control in both DPPH and ABTS assay. (a) Ascorbic acid has maximum scavenging activity from 7.81 μg/ml to 1000 μg/ml in constant manner. (b) Minimum scavenging activity was found at 15.62 μg/ml and it was increased in concentration dependent manner. All values are expressed as Mean ± S.E.M (*n* = 4/group). (TIFF 6.90 mb)

Additional file 5: **Figure S4.** HPLC data for the SOE and identified compound. (TIFF 1.47 mb)

Additional file 6: **Figure S5.** 3D images of femoral condyle bone obtained from Micro-CT and their parameters. (TIFF 9.40 mb)

Additional file 7: **Figure S6**. On the basis of molecular changes, histology, and micro-CT, it is concluded that SOE shows chondro-protective effects on subchondral bone and causes the shifting of chondrocytes and cartilage homeostasis towards anabolism. (TIFF 709 kb)

## Abbreviations

ALT: Alanine amino-transferase
AST: Aspartate amino-transferase
BMP2: Bone morphogenetic protein 2 (Fig. 6B)
Col10: Collagen type 10
Col2: Collagen type-II
COMP: Cartilage oligomeric matrix protein
CTX-II: C-telopeptide of type II collagen
GSTs: Glutathione S-transferases
IL-1β: Interleukin-1 beta
MIA: Monosodium iodoacetate
MMP-1: Matrix metalloproteinases like interstitial collagenase
MMP-13: Matrix metalloproteinases collagenase 3
MMP-3: Matrix metalloproteinases stromelysin-1
MMPs: Matrix metalloproteinases
OA: Osteoarthritis
ROS: Reactive oxygen species
SOE: *Spinacia oleracea* extract
SOX9: Sex determining region Y-box 9 (Fig. 6A)
TIMPs: Tissue inhibitors of matrix metalloproteinases
TNFα: Tumor necrosis factor alpha

## References

[1] Bhatia D, Bejarano T, Novo M (2013). Current interventions in the management of knee osteoarthritis. J Pharm Bioallied Sci.

[2] Pal CP, Singh P, Chaturvedi S, Pruthi KK, Vij A (2016). Epidemiology of knee osteoarthritis in India and related factors. Indian J Orthop.

[3] Zhang W, Ouyang H, Dass CR, Xu J (2016). Current research on pharmacologic and regenerative therapies for osteoarthritis. Bone Res.

[4] Sharma AR, Jagga S, Lee SS, Nam JS (2013). Interplay between cartilage and subchondral bone contributing to pathogenesis of osteoarthritis. Int J Mol Sci.

[5] Sancho-Tello M, Forriol F, Gastaldi P, Ruiz-Sauri A, Martin de Llano JJ, Novella-Maestre E, Antolinos-Turpin CM, Gomez-Tejedor JA, Gomez Ribelles JL, Carda C (2015). Time evolution of in vivo articular cartilage repair induced by bone marrow stimulation and scaffold implantation in rabbits. Int J Artif Organs.

[6] Zhen G, Wen C, Jia X, Li Y, Crane JL, Mears SC, Askin FB, Frassica FJ, Chang W, Yao J (2013). Inhibition of TGF-beta signaling in mesenchymal stem cells of subchondral bone attenuates osteoarthritis. Nat Med.

[7] Chen Y, Sun Y, Pan X, Ho K, Li G (2015). Joint distraction attenuates osteoarthritis by reducing secondary inflammation, cartilage degeneration and subchondral bone aberrant change. Osteoarthr Cartil.

[8] Reed KN, Wilson G, Pearsall A, Grishko VI (2014). The role of mitochondrial reactive oxygen species in cartilage matrix destruction. Mol Cell Biochem.

[9] Nasri H, Baradaran A, Shirzad H, Rafieian-Kopaei M (2014). New concepts in nutraceuticals as alternative for pharmaceuticals. Int J Prev Med.

[10] Gil MI, Ferreres F, Tomas-Barberan FA (1999). Effect of postharvest storage and processing on the antioxidant constituents (flavonoids and vitamin C) of fresh-cut spinach. J Agric Food Chem.

[11] Ko SH, Park JH, Kim SY, Lee SW, Chun SS, Park E (2014). Antioxidant effects of spinach (Spinacia Oleracea L.) supplementation in Hyperlipidemic rats. Prev Nutr Food Sci.

[12] Breitbart E, Lomnitski L, Nyska A, Malik Z, Bergman M, Sofer Y, Haseman JK, Grossman S (2001). Effects of water-soluble antioxidant from spinach, NAO, on doxorubicin-induced heart injury. Hum Exp Toxicol.

[13] Joseph JA, Shukitt-Hale B, Denisova NA, Bielinski D, Martin A, McEwen JJ, Bickford PC (1999). Reversals of age-related declines in neuronal signal transduction, cognitive, and motor behavioral deficits with blueberry, spinach, or strawberry dietary supplementation. J Neurosci.

[14] Seidlova-Wuttke D, Jarry H, Wuttke W (2013). Plant derived alternatives for hormone replacement therapy (HRT). Horm Mol Biol Clin Investig.

[15] Maeda N, Hada T, Murakami-Nakai C, Kuriyama I, Ichikawa H, Fukumori Y, Hiratsuka J, Yoshida H, Sakaguchi K, Mizushina Y (2005). Effects of DNA polymerase inhibitory and antitumor activities of lipase-hydrolyzed glycolipid fractions from spinach. J Nutr Biochem.

[16] Adhikary S, Choudhary D, Ahmad N, Kumar S, Dev K, Mittapelly N, Pandey G, Mishra PR, Maurya R, Trivedi R (2017). Dried and free flowing granules of Spinacia Oleracea accelerate bone regeneration and alleviate postmenopausal osteoporosis. Menopause.

[17] Kim WK, Chung HJ, Pyee Y, Choi TJ, Park HJ, Hong JY, Shin JS, Lee JH, Ha IH, Lee SK (2016). Effects of intra-articular SHINBARO treatment on monosodium iodoacetate-induced osteoarthritis in rats. Chin Med.

[18] Zhang X, Siclari VA, Lan S, Zhu J, Koyama E, Dupuis HL, Enomoto-Iwamoto M, Beier F, Qin L (2011). The critical role of the epidermal growth factor receptor in endochondral ossification. J Bone Miner Res.

[19] Vijayarathna S, Sasidharan S (2012). Cytotoxicity of methanol extracts of Elaeis Guineensis on MCF-7 and Vero cell lines. Asian Pac J Trop Biomed.

[20] Sashidhara KV, Modukuri RK, Choudhary D, Bhaskara Rao K, Kumar M, Khedgikar V, Trivedi R (2013). Synthesis and evaluation of new coumarin-pyridine hybrids with promising anti-osteoporotic activities. Eur J Med Chem.

[21] Lu Y, Xue Y, Chen S, Zhu H, Zhang J, Li XN, Wang J, Liu J, Qi C, Du G (2016). Antioxidant Lignans and Neolignans from Acorus tatarinowii. Sci Rep.

[22] Saeed N, Khan MR, Shabbir M (2012). Antioxidant activity, total phenolic and total flavonoid contents of whole plant extracts Torilis Leptophylla L. BMC Complement Altern Med.

[23] Loganayaki N, Siddhuraju P, Manian S (2013). Antioxidant activity and free radical scavenging capacity of phenolic extracts from Helicteres isora L. and Ceiba Pentandra L. J Food Sci Technol.

[24] Yadav NK, Arya RK, Dev K, Sharma C, Hossain Z, Meena S, Arya KR, Gayen JR, Datta D: Alcoholic Extract of *Eclipta alba* Shows In Vitro Antioxidant and Anticancer Activity without Exhibiting Toxicological Effects. Oxid Med Cell Longev. 2017;2017:9094641. 10.1155/2017/9094641 PMC5307245 28250894

[25] Janero DR (1990). Malondialdehyde and thiobarbituric acid-reactivity as diagnostic indices of lipid peroxidation and peroxidative tissue injury. Free Radic Biol Med.

[26] Wang WH, Tyan YC: Evaluation of the antioxidant activity and antiproliferative effect of the jaboticaba (*Myrciaria cauliflora*) seed extracts in oral carcinoma cells. Biomed Res Int. 2014;2014:185946. 10.1155/2014/185946 PMC4150497 25197631

[27] Zhou PH, Ma BL, Shi L, Xie T, Qiu B (2015). Inhibition of interleukin-1beta-stimulated matrix metalloproteinases via the controlled release of interleukin-1Ra from chitosan microspheres in chondrocytes. Mol Med Rep.

[28] Williams A, Smith JR, Allaway D, Harris P, Liddell S, Mobasheri A (2013). Carprofen inhibits the release of matrix metalloproteinases 1, 3, and 13 in the secretome of an explant model of articular cartilage stimulated with interleukin 1beta. Arthritis Res Ther.

[29] Khedgikar V, Kushwaha P, Gautam J, Sharma S, Verma A, Choudhary D, Mishra PR, Trivedi R (2016). Kaempferol targets Krt-14 and induces cytoskeletal mineralization in osteoblasts: a mechanistic approach. Life Sci.

[30] Bove SE, Calcaterra SL, Brooker RM, Huber CM, Guzman RE, Juneau PL, Schrier DJ, Kilgore KS (2003). Weight bearing as a measure of disease progression and efficacy of anti-inflammatory compounds in a model of monosodium iodoacetate-induced osteoarthritis. Osteoarthr Cartil.

[31] Ekici AG, Akyol O, Ekici M, Sitilci T, Topacoglu H, Ozyuvaci E (2014). Intra-articular injection of dexketoprofen in rat knee joint: histopathologic assessment of cartilage & synovium. Indian J Med Res.

[32] Schmitz N, Laverty S, Kraus VB, Aigner T (2010). Basic methods in histopathology of joint tissues. Osteoarthr Cartil.

[33] Choudhary D, Pandey A, Adhikary S, Ahmad N, Bhatia C, Bhambhani S, Trivedi PK, Trivedi R (2016). Genetically engineered flavonol enriched tomato fruit modulates chondrogenesis to increase bone length in growing animals. Sci Rep.

[34] Jeong YJ, Kim I, Cho JH, Park DW, Kwon JE, Jung MW, Meng X, Jo SM, Song HS, Cho YM (2015). Anti-Osteoarthritic effects of the Litsea Japonica fruit in a rat model of osteoarthritis induced by monosodium Iodoacetate. PLoS One.

[35] Khedgikar V, Ahmad N, Kushwaha P, Gautam J, Nagar GK, Singh D, Trivedi PK, Mishra PR, Sangwan NS, Trivedi R (2015). Preventive effects of withaferin a isolated from the leaves of an Indian medicinal plant Withania Somnifera (L.): comparisons with 17-beta-estradiol and alendronate. Nutrition.

[36] Jeong JH, Moon SJ, Jhun JY, Yang EJ, Cho ML, Min JK (2015). Eupatilin exerts Antinociceptive and Chondroprotective properties in a rat model of osteoarthritis by Downregulating oxidative damage and catabolic activity in Chondrocytes. PLoS One.

[37] Singh S, Mishra A, Shukla S: ALCAR Exerts Neuroprotective and Pro-Neurogenic Effects by Inhibition of Glial Activation and Oxidative Stress via Activation of the Wnt/beta-Catenin Signaling in Parkinsonian Rats. Mol Neurobiol. 2016;53(7):4286–301. 10.1007/s12035-015-9361-5 26223802

[38] Sundaram MS, Hemshekhar M, Santhosh MS, Paul M, Sunitha K, Thushara RM, NaveenKumar SK, Naveen S, Devaraja S, Rangappa KS (2015). Tamarind seed (Tamarindus Indica) extract ameliorates adjuvant-induced arthritis via regulating the mediators of cartilage/bone degeneration, inflammation and oxidative stress. Scientific reports.

[39] Di Cesare ML, Micheli L, Zanardelli M, Ghelardini C (2013). Low dose native type II collagen prevents pain in a rat osteoarthritis model. BMC Musculoskelet Disord.

[40] Bai B, Li Y (2016). Combined detection of serum CTX-II and COMP concentrations in osteoarthritis model rabbits: an effective technique for early diagnosis and estimation of disease severity. J Orthop Surg Res.

[41] Choudhary D, Kushwaha P, Gautam J, Kumar P, Verma A, Kumar A, Maurya SW, Siddiqui IR, Mishra PR, Maurya R (2016). Fast and long acting neoflavonoids dalbergin isolated from Dalbergia Sissoo heartwood is osteoprotective in ovariectomized model of osteoporosis: Osteoprotective effect of Dalbergin. Biomed Pharmacother.

[42] Houard X, Goldring MB, Berenbaum F (2013). Homeostatic mechanisms in articular cartilage and role of inflammation in osteoarthritis. Curr Rheumatol Rep.

[43] Ziskoven C, Jager M, Zilkens C, Bloch W, Brixius K, Krauspe R (2010). Oxidative stress in secondary osteoarthritis: from cartilage destruction to clinical presentation?. Orthop Rev.

[44] Mobasheri A (2013). The future of osteoarthritis therapeutics: targeted pharmacological therapy. Curr Rheumatol Rep.

[45] Mobasheri A (2012). Intersection of inflammation and herbal medicine in the treatment of osteoarthritis. Curr Rheumatol Rep.

[46] Kim JK, Park SW, Kang JW, Kim YJ, Lee SY, Shin J, Lee S, Lee SM: Effect of GCSB-5, a Herbal Formulation, on Monosodium Iodoacetate-Induced Osteoarthritis in Rats. Evid Based Complement Alternat Med. 2012;2012:730907. 10.1155/2012/730907 PMC3303749 22474519

[47] Wang ZM, Chen YC, Wang DP (2016). Resveratrol, a natural antioxidant, protects monosodium iodoacetate-induced osteoarthritic pain in rats. Biomed Pharmacother.

[48] Jeong JW, Kim J, Choi EO, Kwon DH, Kong GM, Choi IW, Kim BH, Kim GY, Lee KW, Kim KY (2017). Schisandrae Fructus ethanol extract ameliorates inflammatory responses and articular cartilage damage in monosodium iodoacetate-induced osteoarthritis in rats. EXCLI J.

[49] Bahtiar A, Nurazizah M, Roselina T, Tambunan AP, Arsianti A (2017). Ethanolic extracts of babandotan leaves (Ageratum Conyzoides L.) prevents inflammation and proteoglycan degradation by inhibiting TNF-alpha and MMP-9 on osteoarthritis rats induced by monosodium iodoacetate. Asian Pac J Trop Med.

[50] Kumari RR, More AS, Gupta G, Lingaraju MC, Balaganur V, Kumar P, Kumar D, Sharma AK, Mishra SK, Tandan SK (2015). Effect of alcoholic extract of Entada Pursaetha DC on monosodium iodoacetate-induced osteoarthritis pain in rats. Indian J Med Res.

[51] Woo YJ, Joo YB, Jung YO, Ju JH, Cho ML, Oh HJ, Jhun JY, Park MK, Park JS, Kang CM (2011). Grape seed proanthocyanidin extract ameliorates monosodium iodoacetate-induced osteoarthritis. Exp Mol Med.

[52] Kim JK, Park SW, Kang JW, Kim YJ, Lee SY, Shin J, Lee S, Lee SM (2012). Effect of GCSB-5, a herbal formulation, on monosodium Iodoacetate-induced osteoarthritis in rats. Evid Based Complement Alternat Med.

[53] Wojdasiewicz P, Poniatowski LA, Szukiewicz D. The role of inflammatory and anti-inflammatory cytokines in the pathogenesis of osteoarthritis. Mediators Inflamm. 2014;2014:561459. 10.1155/2014/561459 PMC4021678 24876674

[54] Goldring MB, Otero M, Plumb DA, Dragomir C, Favero M, El Hachem K, Hashimoto K, Roach HI, Olivotto E, Borzi RM (2011). Roles of inflammatory and anabolic cytokines in cartilage metabolism: signals and multiple effectors converge upon MMP-13 regulation in osteoarthritis. Eur Cell Mater.

[55] Dai L, Zhang X, Hu X, Zhou C, Ao Y (2012). Silencing of microRNA-101 prevents IL-1beta-induced extracellular matrix degradation in chondrocytes. Arthritis Res Ther.

