# Supplementary figures and images for: Spinacia oleracea extract attenuates disease progression and sub-chondral bone changes in monosodium iodoacetate-induced osteoarthritis in rats
Source: BMC Complement Altern Med. 2018 Feb 20;18:69. doi: 10.1186/s12906-018-2117-9 (PMC5819303; doi:10.1186/s12906-018-2117-9)

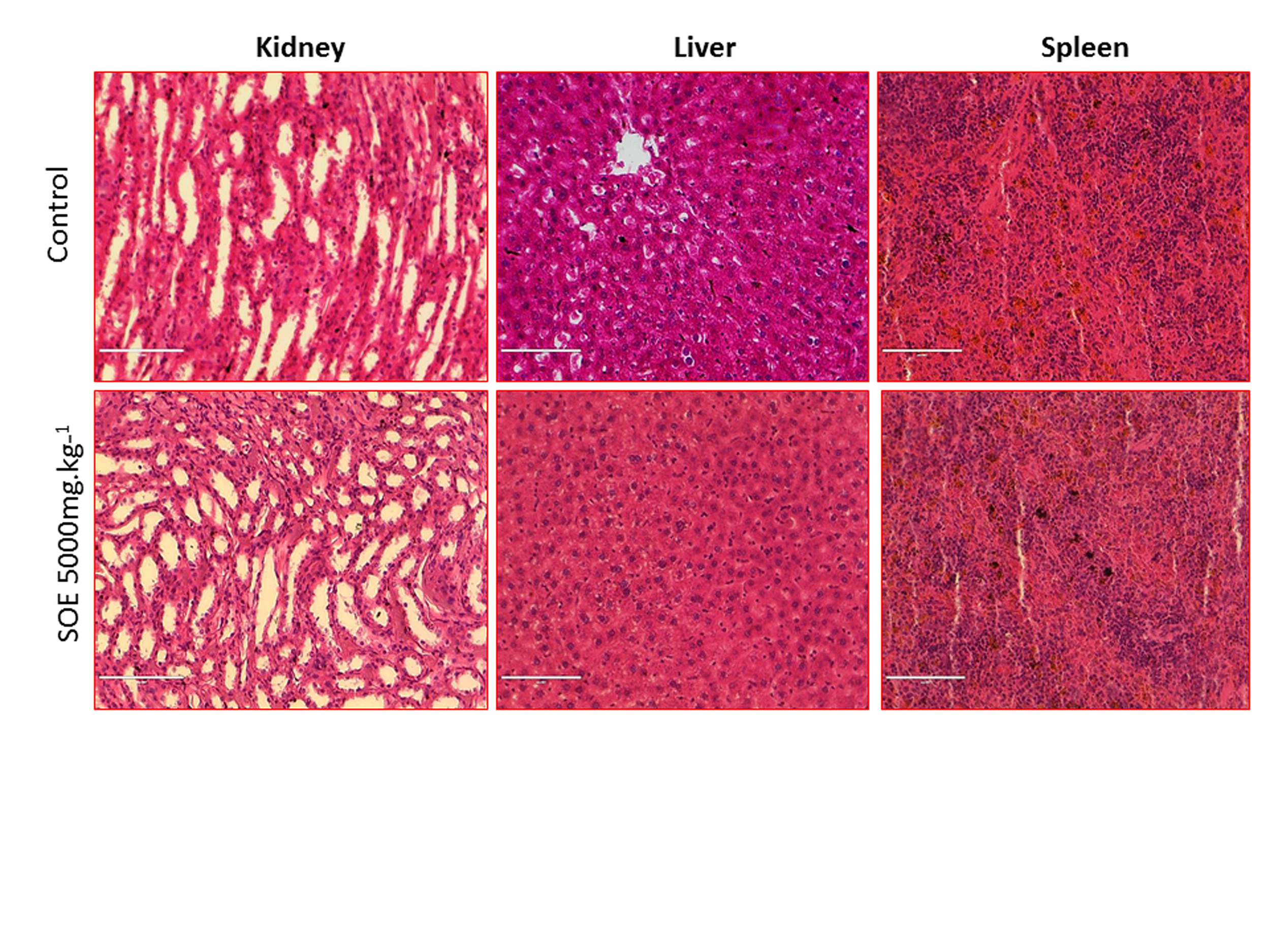

Supplement: Supplementary file 1 — Figure S1. H&E stained organ sections, isolated from rat after treatment of SOE (acute toxicity study). No noticeable abnormality was observed in major organs including kidney, liver, and spleen. (TIFF 9629 kb) [file 12906_2018_2117_MOESM1_ESM.tif]

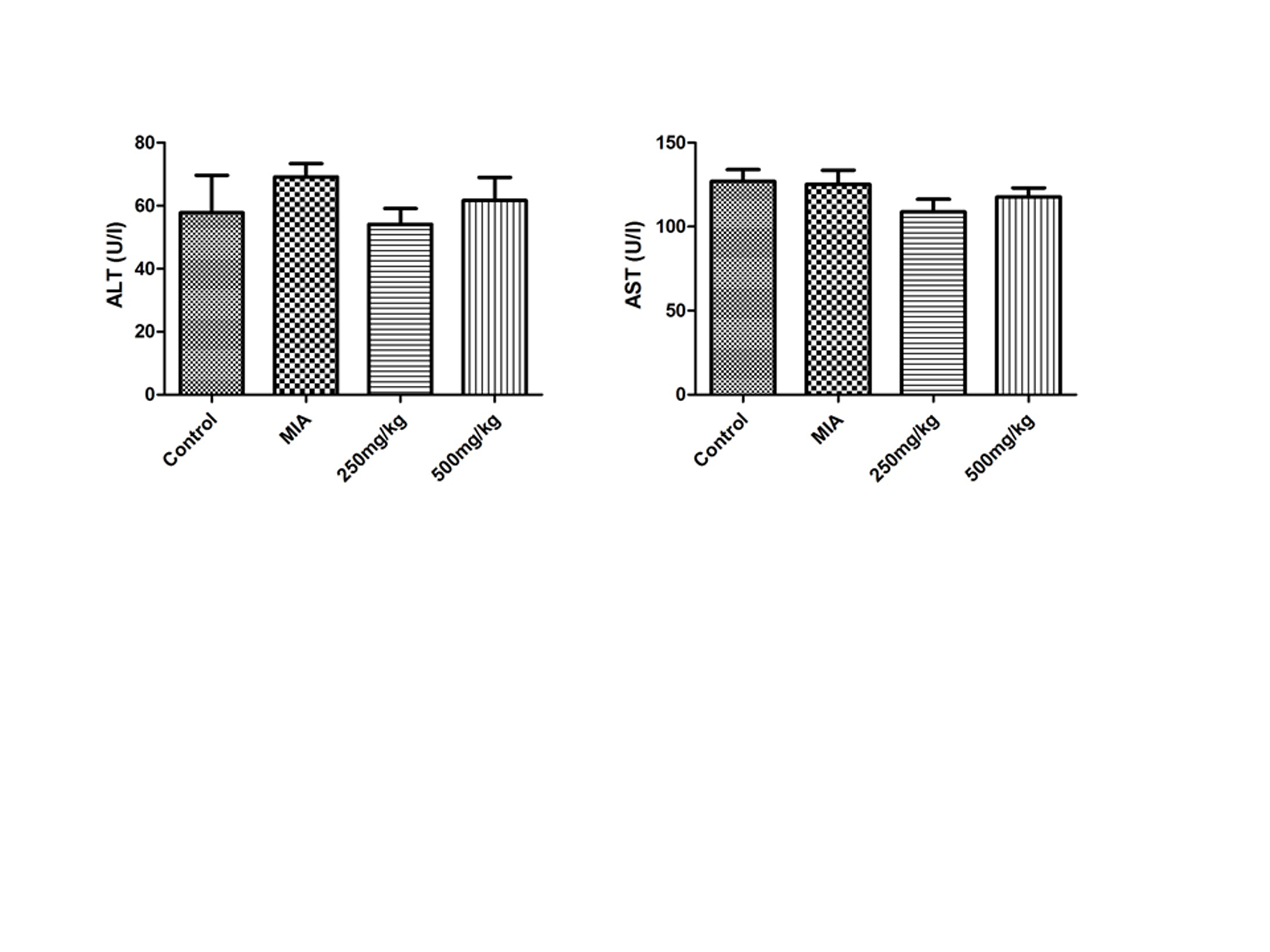

Supplement: Supplementary file 2 — Figure S2. Serum ALT and AST level in different groups after 28 days of treatment of SOE. No significant differences in control, MIA, 250 mg/kg, 500 mg/kg. All values are expressed as Mean ± S.E.M (n = 4/group). (TIFF 7976 kb) [file 12906_2018_2117_MOESM2_ESM.tif]

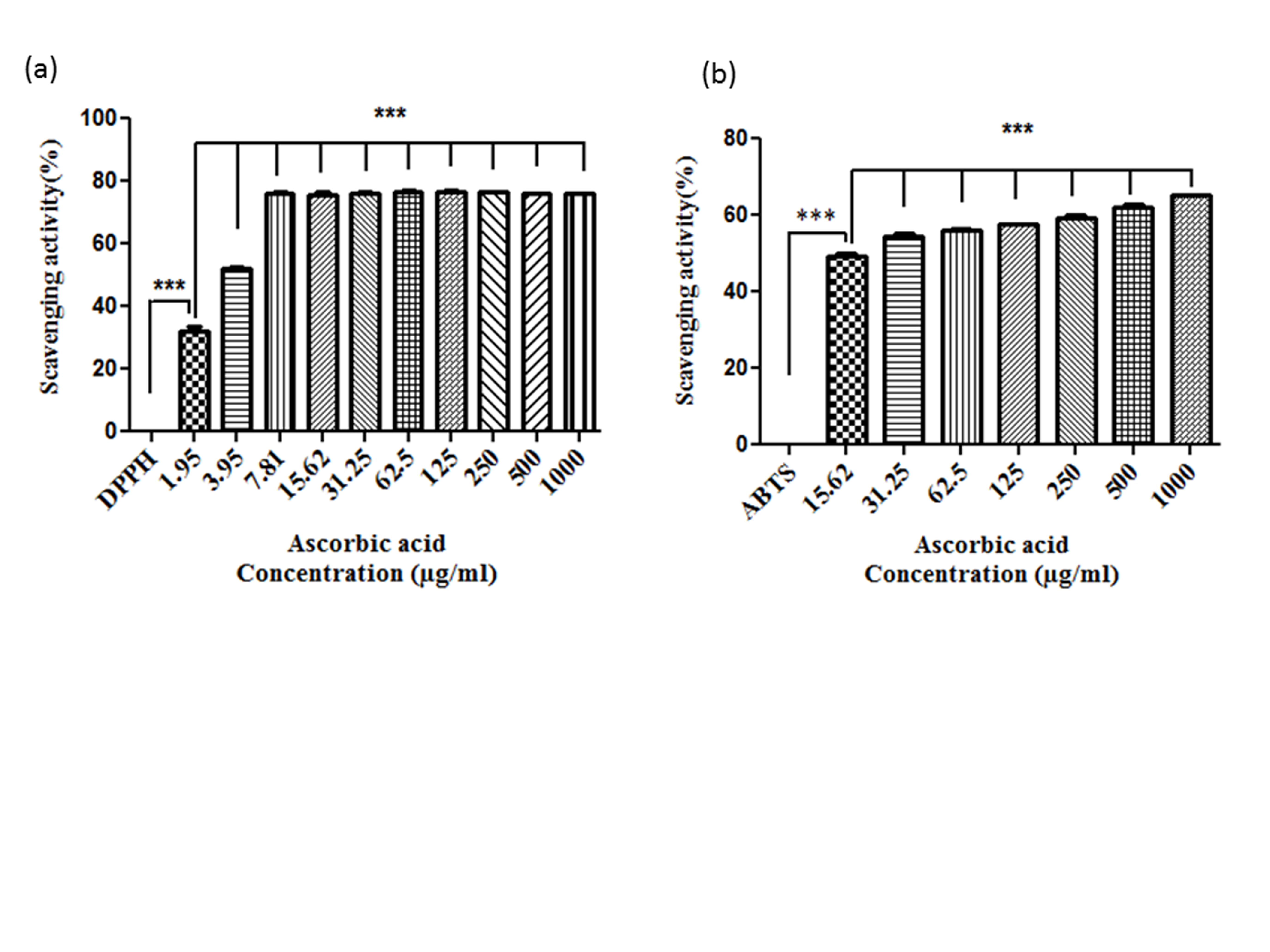

Supplement: Supplementary file 4 — Figure S3. Ascorbic acid was used as positive control in both DPPH and ABTS assay. (a) Ascorbic acid has maximum scavenging activity from 7.81 μg/ml to 1000 μg/ml in constant manner. (b) Minimum scavenging activity was found at 15.62 μg/ml and it was increased in concentration dependent manner. All values are expressed as Mean ± S.E.M (n = 4/group). (TIFF 6.90 mb) [file 12906_2018_2117_MOESM4_ESM.tif]

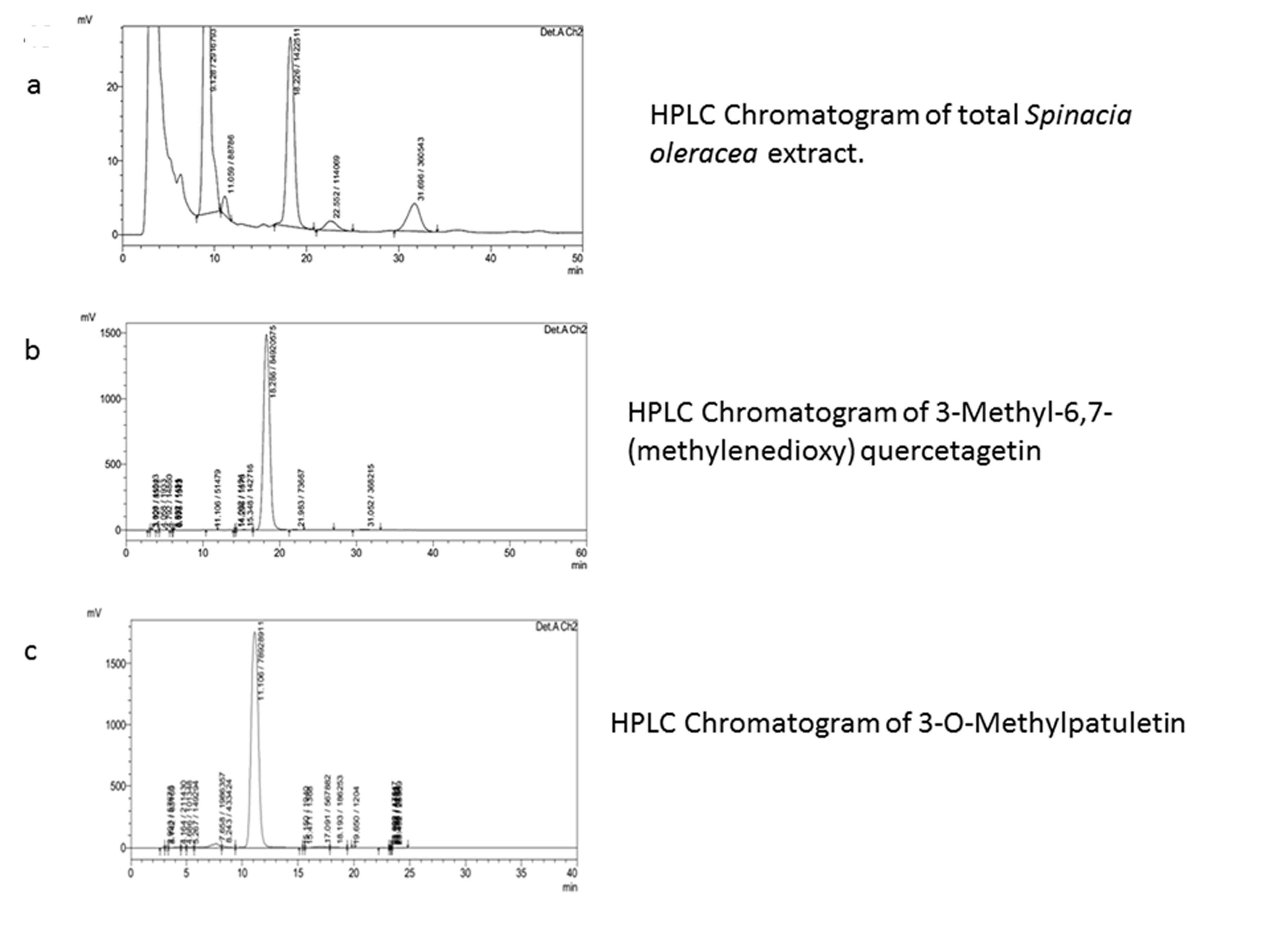

Supplement: Supplementary file 5 — Figure S4. HPLC data for the SOE and identified compound. (TIFF 1.47 mb) [file 12906_2018_2117_MOESM5_ESM.tif]

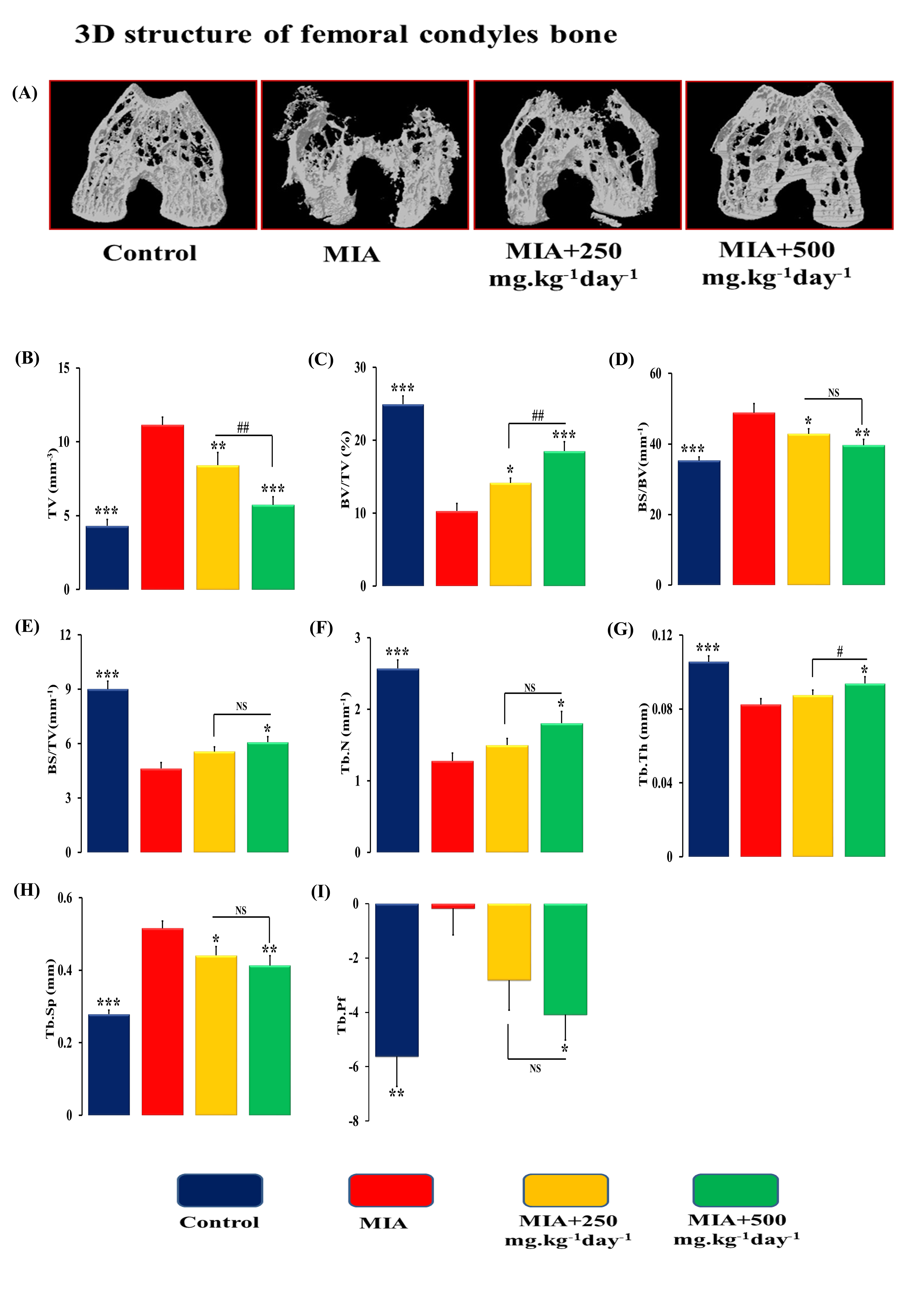

Supplement: Supplementary file 6 — Figure S5. 3D images of femoral condyle bone obtained from Micro-CT and their parameters. (TIFF 9.40 mb) [file 12906_2018_2117_MOESM6_ESM.tif]

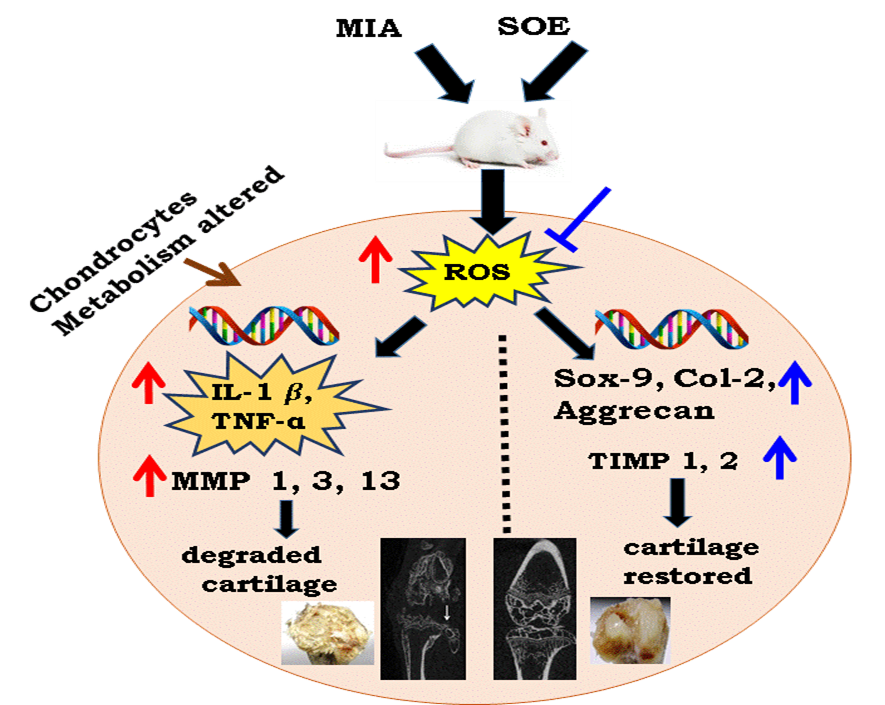

Supplement: Supplementary file 7 — Figure S6. On the basis of molecular changes, histology, and micro-CT, it is concluded that SOE shows chondro-protective effects on subchondral bone and causes the shifting of chondrocytes and cartilage homeostasis towards anabolism. (TIFF 709 kb) [file 12906_2018_2117_MOESM7_ESM.tif]
