# Supplementary material for: Spinacia oleracea extract attenuates disease progression and sub-chondral bone changes in monosodium iodoacetate-induced osteoarthritis in rats
Source: BMC Complement Altern Med. 2018 Feb 20;18:69. doi: 10.1186/s12906-018-2117-9 (PMC5819303; doi:10.1186/s12906-018-2117-9)
Supplement: Supplementary file 3 — Supplementary information. (DOCX 16.5 kb) [file 12906_2018_2117_MOESM3_ESM.docx]

**Materials and Methods**

**H and E Staining**

5g/kg dose of SOE given to female SD rat (n=5). After 15 days rats were euthanized and kidney, liver, spleen were collected in 4% formaldehyde. Organs were fixed in paraffin wax and 5.0µm sectionwere cut by leica rotatory microtome. Sections were further stained with haematoxylin and eosin. Representative images were captured by EOVS XL, Life technologies.^1^

**ALT and AST Assay**

For the evaluation of biochemical parameters, one aliquot of blood per animal was placed in a tube and serum was isolated. Serum aliquots were subjected to evaluation of: alanine amino-transferase (ALT), aspartate amino-transferase (AST). All parameters were performed using Selectra junior fully automatic biochemical analyser according to manufacturer’s protocol.^2^

**Results:**

**SOE treatment has no toxic effect**

We further studied the toxic effects of SOE on kidney, liver and spleen. We found that SOE has no toxic effects on on major organs (Figure S2).

**SOE treatment has no effect on Liver enzymes (AST and ALT)**

Liver Enzymes, AST and ALT, were assessed in serum and no significant changes has shown in Control, MIA, 250mg/kg, 500mg/kg doses (Figure S5)

**References**

1. Please refer reference no. 35 of main manuscript.

2. Majhi S, Baral N, Lamsal M, Mehta KD. De Ritis rati as diagnostic marker of alcoholic liver disease. Nepal Med Coll J. 2006: 8(1):40-2.

**Figure legends for Supplementary data**

**Figure S1:** H&E stained organ sections, isolated from rat after treatment of SOE (acute toxicity

study). No noticeable abnormality was observed in major organs including kidney,

liver, and spleen.

**Figure S2:** Serum ALT and AST level in different groups after 28 days of treatment of SOE. No significant differences in control, MIA, 250mg/kg, 500mg/kg. All values are expressed as Mean ± S.E.M (n=4/group).

.

**Figure S3:** Ascorbic acid was used as positive control in both DPPH and ABTS assay. (a) Ascorbic acid has maximum scavenging activity from 7.81µg/ml to 1000µg/ml in constant manner. (b) Minimum scavenging activity was found at 15.62µg/ml and it was increased in concentration dependent manner. All values are expressed as Mean ± S.E.M (n=4/group).

**Figure S4:** HPLC data for the SOE and identified compound

**Figure S5:** 3D images of femoral condyle bone obtained from Micro-CT and their parameters

**Figure S6:** On the basis of molecular changes, histology, and micro-CT, it is concluded that SOE shows chondro-protective effects on subchondral bone and causes the shifting of chondrocytes and cartilage homeostasis towards anabolism.
